# Non-emergency department (ED) interventions to reduce ED utilization: a scoping review

**DOI:** 10.1186/s12873-024-01028-4

**Published:** 2024-07-12

**Authors:** Målfrid A. Nummedal, Sarah King, Oddvar Uleberg, Sindre A. Pedersen, Lars Petter Bjørnsen

**Affiliations:** 1 Trondheim Emergency Department Research Group (TEDRG), Department of Circulation and Medical Imaging, Faculty of Medicine and Health Sciences, Norwegian University of Science and Technology (NTNU), Trondheim, Norway; 2 Clinic of Emergency Medicine and Prehospital Care, St. Olav’s Hospital, Trondheim University Hospital, Trondheim, Norway; 3 Department of Research and Development, Division of Emergencies and Critical Care, Oslo University Hospital, Oslo, Norway; 4 The Medicine and Health Library, Library Section for Research Support, Data and Analysis, Norwegian University of Science and Technology (NTNU), Trondheim, Norway

**Keywords:** ED crowding, ED utilization, Patient influx

## Abstract

**Background:**

Emergency department (ED) crowding is a global burden. Interventions to reduce ED utilization have been widely discussed in the literature, but previous reviews have mainly focused on specific interventions or patient groups within the EDs. The purpose of this scoping review was to identify, summarize, and categorize the various types of non-ED-based interventions designed to reduce unnecessary visits to EDs.

**Methods:**

This scoping review followed the JBI Manual for Evidence Synthesis and the PRISMA-SCR checklist. A comprehensive structured literature search was performed in the databases MEDLINE and Embase from 2008 to March 2024. The inclusion criteria covered studies reporting on interventions outside the ED that aimed to reduce ED visits. Two reviewers independently screened the records and categorized the included articles by intervention type, location, and population.

**Results:**

Among the 15,324 screened records, we included 210 studies, comprising 183 intervention studies and 27 systematic reviews. In the primary studies, care coordination/case management or other care programs were the most commonly examined out of 15 different intervention categories. The majority of interventions took place in clinics or medical centers, in patients’ homes, followed by hospitals and primary care settings - and targeted patients with specific medical conditions.

**Conclusion:**

A large number of studies have been published investigating interventions to mitigate the influx of patients to EDs. Many of these targeted patients with specific medical conditions, frequent users and high-risk patients. Further research is needed to address other high prevalent groups in the ED - including older adults and mental health patients (who are ill but may not need the ED). There is also room for further research on new interventions to reduce ED utilization in low-acuity patients and in the general patient population.

**Supplementary Information:**

The online version contains supplementary material available at 10.1186/s12873-024-01028-4.

## Introduction

Emergency Department (ED) crowding is a global public health issue [1]. The increased number of patient visits to the ED has negative consequences for patients, staff, and the entire healthcare system [2, 3]. ED crowding is a complex and multifactorial problem, often attributed to factors such as the growing complexity of patient conditions, an aging population, and limited healthcare accessibility [3].

While many intervention strategies have focused on improving processes within the ED itself [4, 5], it is evident that addressing the causes of ED crowding requires a broader approach encompassing the larger healthcare system [3]. For example, exit (or access) block in EDs is often a problem due to lack of hospital beds or other facilities to send patients to [6]. Researchers have also considered non-ED interventions as a promising avenue for reducing ED visits and mitigating crowding. These interventions primarily target upstream settings including primary care [7, 8], community health [9], and the redirection of low-acuity patients to other healthcare services [10]. The overarching goal is to enhance access to appropriate care in these settings, preventing unnecessary ED visits and ensuring care is directed to the most suitable location. Other strategies are to monitor patients, educate them about their health and empower them with self-management strategies to prevent health condition escalation. In addition, case management interventions - a collaborative approach aimed at addressing specific patient needs - has been extensively tested and evaluated [11, 12].

Previous reviews of non-ED interventions have focused on specific patient groups [13–15] or specific categories of interventions [7, 12]. While Morgan et al. [16] conducted a comprehensive systematic review of non-ED interventions in 2013, their review excluded case management and telephone triage due to previously conducted systematic reviews on those topics [13, 17]. Given continuous changes in healthcare and demand, and the publication of numerous studies since then, an updated summary of all types of non-ED interventions aiming to reduce unnecessary ED visits was warranted.

The objective of this scoping review was to map the literature for non-ED interventions implemented in the last c. 15 years (from 2008 to early 2024) that aimed to mitigate the influx of patients into the ED. We aimed to provide an up-to-date overview and to identify potential knowledge gaps.

## Methods

### Search strategy

We conducted the scoping review according to the principles presented in the JBI Manual for Evidence Synthesis [18] and followed the criteria set out in the PRISMA-ScR checklist [19]. The protocol is available upon request from the study authors. Given that no human subjects or medical records were reviewed as a part of this study, approval from a human research ethics committee was not required. We reviewed the literature for studies reporting on logistical non-ED interventions (i.e., outside of the ED) aimed at reducing ED use.

With the assistance of a medical research librarian (SAP), a comprehensive structured search was performed in the databases MEDLINE and Embase from 2008 to January 2023, and updated on the 30th March, 2024. The search was based on thesaurus- and free-text terms for the three main concepts ‘EDs’, ‘visits’ and ‘interventions to anticipate and reduce visits’. Details on the search strategy used in the databases is available in Additional file 1.

### Inclusion and exclusion criteria

Studies that examined interventions in non-ED settings that specifically aimed to reduce or control ED patient influx as a primary or secondary objective were eligible for inclusion. Hospital based interventions were eligible for inclusion as long as the intervention did not require ED staff or resources. Studies that evaluated changes in medication or treatment were not included. In addition, studies that examined the effects of broader health system changes or reforms were excluded. The interventions could be aimed at the general population or target specific patient groups. Eligible study designs included randomized controlled trials (RCTs), non-randomized controlled studies, cohort studies, pre-post studies, as well as systematic reviews (SRs). They could be from any country and in any language, but had to be published within the last c.15 years to encompass the most recent period of patient demand. The studies had to clearly report a measure of ED utilization (e.g., number of ED visits) using hospital data; studies that presented self-reported ED visits were excluded. Studies reported as abstracts were only included if adequate data were reported within them and if there was no full paper associated with the abstract. Initially, we aimed to include studies that evaluated the use of prediction or forecasting models to effectively manage resources to cope with ED influx (but not methodology papers). During the screening phase, only methodological papers were found. This topic area in general was considered to be a different concept that did not measure ED utilization and was omitted from this scoping review.

### Study selection and data extraction

Four reviewers worked in two pairs (MAN and SEK, LPB and OU) to screen the titles and abstracts. Two reviewers (MAN and SEK) independently screened the full texts for inclusion. Disagreements were resolved by group discussion. Study characteristics were extracted by one reviewer and checked by another (MAN and SEK). Details on study design, population and intervention methods were data extracted from the intervention studies. Results were not extracted as we did not aim to examine effectiveness, but to map the evidence. For the SRs, information was extracted on intervention and population inclusion criteria, the SR primary and secondary outcomes, the number and type of studies included, the types of non-ED interventions included in the SR, and the SR conclusions. If both ED and non-ED interventions were presented in a SR, only the non-ED interventions were extracted.

### Categorization of the data

To enable mapping of the data, the intervention studies were broadly categorized by population group (e.g. general population, or specific patient groups), age, as well as type and location of the intervention. The categorization of intervention types were made through reviewer consensus (MAN, SEK, LPB and OU) after the data extraction. For the SRs, categorizations made by the SR authors were used where possible.

## Results

**Fig. 1:**
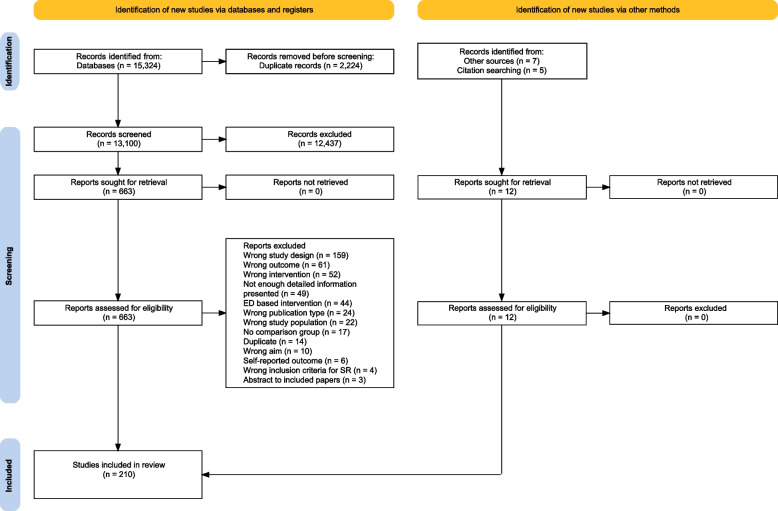
Prisma flow diagram [20] of the inclusion and exclusion process

In total, 15,324 records were identified in the search. After removing duplicates, 13,100 titles and abstracts were screened and 12,437 were deemed irrelevant to the topic area. Of the remaining 663 papers, 198 met the inclusion criteria and another 12 studies were included from citation searching ($n=5$) and other sources (e.g., communication with topic experts) ($n=7$) for a total of 210 included studies (see Fig. 1 for Prisma flow diagram).

### Study characteristics

The publication dates of the included studies spanned from 2008 to 2024, with 164 (78%) published the last 10 years. The eligible studies included before-and-after studies ($n=93$), controlled trials (randomized and non-randomized) ($n=49$), retrospective and prospective cohorts ($n=30$), time series ($n=9$), retrospective case control ($n=1$) and a comparative study ($n=1$). The remaining included studies were systematic reviews ($n=27$).

The majority of the intervention studies were conducted in the USA (69%). Twelve were conducted each in Canada and the UK, seven in Spain, three in each Australia, France, Singapore and The Netherlands, two in Mexico, and one in Hong Kong, New Zealand, Sweden, Finland, Switzerland, Romania, Italy, Ireland, Germany, China, Denmark and Taiwan. Additional files 2 and 3 shows the complete data extraction with characteristics of the intervention studies and the systematic reviews, respectively.

### Intervention studies ($n=183$)

#### Interventions

Details of each study are reported in Additional file 2. One-hundred-and-eighty-three studies evaluated 187 interventions. These interventions were broadly categorized into 15 different types based on the most predominant component: care coordination/case management or other care programs ($n=85$) [21–104], capacity increase in non-ED settings ($n=23$) [105–113, 113–126], education ($n=23$), [127–149], follow-up of patients (i.e., from primary care or after an ED or primary care visits or after hospital discharge) ($n=18$) [150–167], telemonitoring ($n=8$) [168–175], structural changes in primary care ($n=6$) [176–182], self-management ($n=5$) [179, 183–186], patient financial incentives ($n=5$) [141, 187–190], pre-hospital triage-type assessments ($n=4$) [141, 191–193], screening tools ($n=3$) [194–196], school based therapy ($n=2$) [197, 198], ED reports to specialists ($n=2$) [199, 200], prevention programs ($n=1$) [201], barrier strategies ($n=1$) [202] and a medication service ($n=1$) [203].

The most frequently assessed interventions were those that involved care coordination/case management or other types of care programs (46%). These interventions were highly diverse and mostly included a form of care coordination. This could be a patient navigator [70], that along with other actions, identified community resources for the patients [27] or led a multidiciplinary team of supportive care [65]. Another intervention that fell within this broad category was case management [21, 23, 24, 29, 46, 53, 54, 56, 57], which is a collaborative approach to meet the patients’ social- and/or health needs using an individual plan. Care programs often consisted of home visits, where health workers perform care, conduct screenings, or help the patients or family with health-related tasks or education of their condition in the patients home [38–41, 44, 45, 47, 49–51, 75, 79, 97–102]. Other care programs involved interdisciplinary teams providing care around different locations and often coordinated care between health sectors [33, 48, 55, 60, 64, 67, 68, 90–93].

The second most commonly reported interventions were education (12%) and capacity increase in non-ED settings (12%). The educational interventions included education of patients alone [143–145], in group sessions [128, 132, 137, 142, 147–149] or educational brochures/posters for the patients [127, 139, 160]. Others were aimed at parents [130, 131, 133, 140], staff [129, 134–136, 148], or for the general public through media [141]. The studies assessing health service capacity increases in non-ED settings included the opening of additional primary care [109, 111, 121, 124] or specialist clinics [107, 108, 110, 112, 113, 119, 120, 122, 123, 125, 126, 141], free primary health clinics [105, 116], increasing primary care hours [114, 115, 118], changing the types of hospital services offered [117] or speed of access to some clinical services [106].

Of the 18 studies examining the impact of follow-up contact, five involved telephone contact after discharge from hospital; [157, 158] three involved a pharmacist [152, 156, 160] and one a physician [154]. Three studies involved telephone contact after discharge from an ED [150, 153, 155] and one a contact at the hospital 1 week after discharge [165]. Two studies involved a follow-up program consisting of a multidisciplinary team [159, 162], and two evalated a specialized clinic for post-discharge contacts [163, 164]. The two last studies involved telephone follow-up of chronic patients by a physician in primary care [151] and a program consisting of home visits, telephone-calls and self-management techniques [166].

Eight studies assessed telemonitoring of patients, either alone [168–171, 175] or in combination with self-management strategies [172–174]. Five studies assessed self-management strategies alone, such as crisis- [183] or action plans [184], a motivating interview to improve medication self-management [179] or different forms of digital content sent to the patient to remind, motivate or help them [185, 186]. Five studies examined the impact of patient financial incentives including an increase in copayment for ED visits [141, 188], free dental care [189] or a one time payment [187] or a ED fee discount [190] if visiting their PCP.

Four studies examined pre-hospital triage-type assessments by healthcare professionals. One was done by a non-ambulance response team [191], another was conducted at the ED door [141] and the two last used a telephone triage system staffed by nurses [192] or an emergency physician [193]. In addition, six studies examined the impact of organizational changes in primary care centres [176–178, 180–182]. These interventions included establishing primary care networks[177], implementing patient-centered medical homes[178, 181], or enhancing primary care services specifically tailored for individuals with serious mental illness [176, 180, 182]. Three studies involved a screening tool, where two were of elderly in a nursing home [194, 195] and the last used augmented intelligence (AI) [196].

The remaining categories, each of which had two or fewer studies, were: school based therapy of asthma patients [197, 198], a medication service [203] which gave patients access to medications at a reduced price, a prevention program [201], barrier strategies with mandatory referral to the ED [202] and ED reports to specialists, where pediatric specialists received reports outlining the rates of ED use by their patients [199, 200].

**Table 1:** Population targeted by which intervention

Populations targeted	Interventions evaluated
General population	**Care coordination/case management or other care programs**
	Care coordination ($n=2$) [63, 66]
	Car coordination and triage ($n=1$) [74]
	EMS care coordination ($n=1$) [36]
	Home visit ($n=2$) [51, 75]
	Medication management ($n=1$) [76]
	**Education**
	Education of staff ($n=4$) [129, 134, 135, 138]
	Education of parents ($n=2$) [133, 140]
	Education of parents and clinic access ($n=1$) [130]
	Education of parents and support ($n=1$) [131]
	Education of patient ($n=1$) [127]
	Education of staff and outreach hospital ($n=1$) [136]
	Public education ($n=1$) [141]
	**Capacity Increase in non-ED settings**
	Additional clinics ($n=2$) [109, 112]
	Increased primary care hours ($n=2$) [114, 115]
	Increased clinic access and care coordination ($n=1$) [122]
	Increased clinic access and education of parents ($n=1$) [123]
	**Structural change in primary care**
	($$n=2$$) [177, 178]
	**Follow-up**
	Telephone follow-up ($n=4$) [150, 155, 157, 158]
	Telephone follow-up with pharmacist ($n=1$) [156]
	Telephone follow-up with physician ($n=1$) [154]
	**Screening tool**
	($$n=2$$) [194, 195]
	**Telemonitoring**
	($$n=1$$) [171]
	**Patient financial incentives**
	($$n=1$$) [141]
	**Triage**
	Telephone triage (using an EP) ($n=1$) [193]
Patients with a specific medical condition^a^	**Care coordination/case management or other care programs**
	Home visit ($n=9$) [39, 44, 45, 47, 50, 97–100]
	Care coordination ($n=9$) [22, 62, 65, 70, 84–88]
	Care program ($n=5$) [30, 48, 55, 90, 91]
	Case management ($n=2$) [21, 54]
	Pharmacist involvement ($n=2$) [52, 61]
	EMS care coordination ($n=1$) [35]
	Home visit and follow-up ($n=1$) [40]
	Medication management ($n=1$) [104]
	Care plan ($n=1$) [89]
	Care model ($n=1$) [70]
	Care program including education, screening and advice ($n=1$) [92]
	Care program including follow-up and education of patient and parents ($n=1$) [93]
	Community paramedic program ($n=1$) [94]
	Health checks ($n=1$) [96]
	Home visit and care coordination ($n=1$) [101]
	Home visit and follow-up ($n=1$) [102]
	Home visit with education ($n=1$) [103]
	**Education**
	Education of patients ($n=10$) [128, 132, 137, 139, 142–146, 149]
	Education of patients and parents ($n=2$) [129, 148]
	Education of staff ($n=1$) [135]
	Education of staff and case management ($n=1$) [129]
	**Capacity increase in non-ED settings**
	Additional clinics ($n=5$) [107, 108, 110, 113, 125]
	Additional services ($n=2$) [117, 126]
	Rapid access to clinical services ($n=1$) [106]
	**Structural change in primary care**
	Structural change in primary care ($n=1$) [176]
	Co-location of clinics ($n=1$) [182]
	**ED reports to specialists**
	($$n=2$$) [199, 200]
	**Patient financial incentives**
	($$n=1$$) [189]
	**Follow up**
	Follow-up program ($n=3$) [162–164]
	Physician follow-up ($n=2$) [151, 165]
	Follow-up with care coordination ($n=1$) [95]
	Follow-up with self-management and care coordination ($n=1$) [166]
	Telephone follow-up and telemonitoring ($n=1$) [167]
	**Prevention program**
	($$n=1$$) [201]
	**School based therapy**
	($$n=2$$) [197, 198]
	**Self-management**
	($n=5$) [179, 183–186]
	**Telemonitoring**
	Telemonitoring and self-management ($n=3$) [172–174]
	Telemonitoring ($n=2$) [170, 175]
	**Screening tool**
	($$n=1$$) [196]
	**Barrier strategies**
	Mandatory referrals ($n=1$) [202]
	**Medication service**
	($$n=1$$) [203]
Frequent users^b^	**Care coordination/case management or other care programs**
	Case management ($n=5$) [23, 24, 53, 56, 57]
	Care coordination ($n=8$) [26, 27, 42, 43, 58, 71–73]
	Care program ($n=3$) [31, 33, 60]
	Community paramedic program ($n=1$)[37]
	EMS care coordination ($n=1$) [34]
	Screening and social program ($n=1$) [25]
	Home visit ($n=1$) [38]
	**Capacity Increase in non-ED settings**
	Free clinics ($n=1$) [116]
	Additional clinics ($n=1$) [121]
	**Telemonitoring**
	($$n=1$$) [169]
High risk patients	**Care coordination/case management or other care programs**
	Care program ($n=3$) [64, 67, 68]
	Case management ($n=2$) [29, 46]
	Care coordination ($n=2$) [77, 78]
	Community program ($n=1$) [32]
	Community paramedic program ($n=1$) [80]
	Medication management ($n=1$) [69]
	Home visit ($n=2$) [49, 79]
	Home visit and follow-up ($n=1$) [41]
	**Follow up**
	Pharmacist follow-up ($n=2$) [152, 160]
	Follow-up program ($n=1$) [159]
	Telephone follow-up ($n=1$) [153]
	**Telemonitoring**
	($$n=1$$) [168]
Homeless^c^	**Capacity increase in non-ED settings**
	Additional clinics ($n=1$) [111]
	**Care coordination/case management or other care programs**
	Care coordination and housing ($n=4$) [28, 81–83]
	**Structural change in primary care**
	Co-location of clinics ($n=1$) [180]
Patients with specific insurance or income status^d^	**Care coordination/case management or other care programs**
	Care coordination ($n=1$) [59]
	**Capacity increase in non-ED settings**
	Free clinics ($n=1$) [105]
	**Patient financial incentives**
	($$n=2$$) [187, 188]
	**Pre-hospital triage-type assessments**
	Telephone triage ($n=1$) [192]
	**Follow-up**
	Telephone follow-up with pharmacist ($n=1$) [161]
	**Structural change in primary-care**
	($$n=1$$) [181]
Low acuity patients	**Capacity increase in non-ED settings**
	Additional clinics ($n=4$) [119, 120, 124, 141]
	Increase primary care hours ($n=1$) [118]
	**Patient financial incentives**
	($$n=1$$) [190]
	**Pre-hospital triage-type assessments**
	Prehospital triage and treatment ($n=2$) [141, 191]

^a^Medical condition includes skin disorders, different cancer diseases, gastro-intestinal diseases, psychological disorders and disabilities, diabetes, chronic conditions, dementia, epilepsy, respiratory conditions, dental conditions, pregnant women, systemic lupus erythematosus, blood disorders, substance abuse, trauma, and heart diseases

^b^Some of the studies reported on frequent users with an additional problem or specified medical condition

^c^Most of the studies included homeless patients with an additional problem or characteristics, such as mental illness, frequent users and veterans

^d^One study reported a patient group with a specific medical condition

#### Location of interventions

**Fig. 2:**
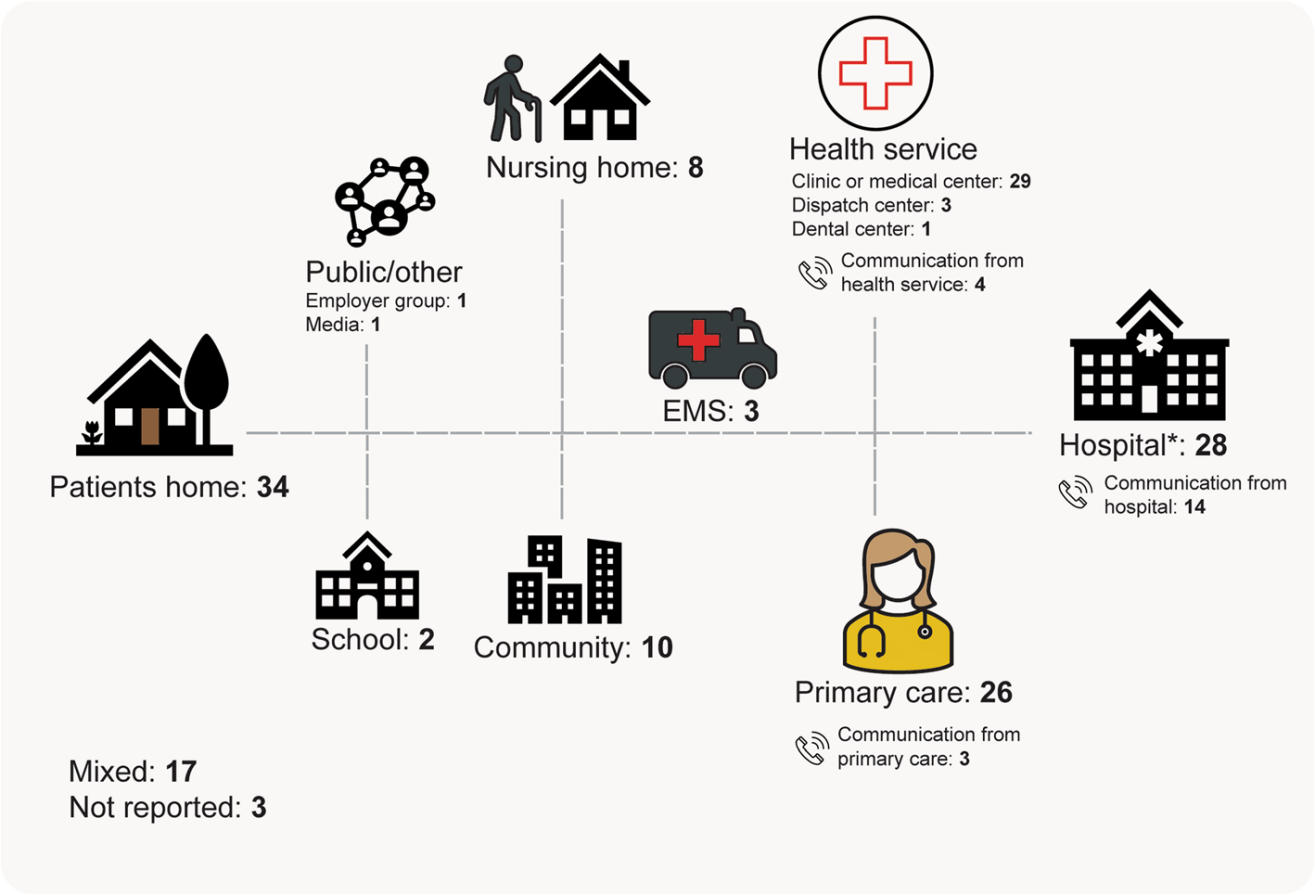
Number of interventions performed by type of location. ‘Mixed’ locations consisted of interventions made up of interdisciplinary teams working in different care facilities. EMS = Emergency Medical Services ^*^Two were based outside the ED front-door

The interventions were conducted in different settings (see Fig. 2). The largest proportions were performed at the patients home ($n=34$) or in a clinic/medical center ($n=29$). The interventions evaluated at home mostly involved a visit ($n=18$) or telemonitoring of patients ($n=6$) (alone or in combination with self-management strategies). Of those performed in a clinic or medical center, most evaluated a capacity increase of the clinic ($n=13$) or care coordination/program or management ($n=9$). Interventions located in primary care ($n=26$) evaluated a large range of interventions, with seven involving a capacity increase such as adding additional clinics ($n=2$) or increased hours ($n=3$). Others involved education ($n=5$), follow-up contacts ($n=3$) or structural change ($n=6$) (intervention involving structural change is described in subsection ‘intervention’). Twenty-eight interventions were hospital based, with two conducted outside the ED front-door - testing pre-hospital triage-type screening with redirection of low-acuity patients and financial disincentives directed at patients attending EDs. The remaining interventions conducted in a hospital were at a specialized department and testing a range of interventions, e.g care- coordination/program or management ($n=9$), education of patients ($n=7$), adding additional services or clinics ($n=3$), a follow-up contact ($n=4$) or receiving reports from the ED about their patients utilization ($n=2$).

Eight studies evaluated an intervention conducted at a nursing home. These interventions were education of staff ($n=2$), care coordination or care program ($n=3$), telemonitoring ($n=1$) and a screening tool to prevent ED visits ($n=2$). Of the three interventions conducted by EMS (Emergency Medicine Services), two were coordination strategies to give the patients access to proper care according to their acuity level, and the last was triage and treatment at the scene by paramedics, physicians, or nurses. Interventions located in the community ($n=10$) were mostly care coordination/case management or other care programs. One study tested a self-management strategy involving an individual crisis plan. The remaining studies were conducted at a dispatch center ($n=3$), at school ($n=2$), a dental center ($n=1$), through media ($n=1$) or an employer group ($n=1$). The studies testing interventions involving communication from hospital ($n=14$), primary care ($n=3$) or a health service ($n=4$) were mostly a telephone follow-up contact after the patient had been discharged from the ED or hospital ($n=11$). Lastly, 17 studies had mixed locations consisting of interventions made up of interdisciplinary teams working in different care facilities, and three studies did not report on the intervention location.

#### Study populations

In terms of populations targeted by the interventions, the majority were aimed at patients with various specific types of medical conditions ($n=89$), followed by frequent ED users ($n=23$) and high risk patients ($n=18$). Among the interventions that focused on patients with predefined medical conditions, individuals with respiratory diseases like asthma or chronic obstructive pulmonary disease ($n=17$) and cancer patients ($n=16$) were the most frequently assessed groups. Interestingly, there were only five intervention studies that specifically targeted patients with mental health issues. Eight intervention studies included low acuity patients. In addition, seven were aimed at patients with a specific insurance or income status, while six studies targeted the homeless. The remaining 36 intervention studies targeted the general population (Table 1).

The majority of studies included all age groups ($n=83$) or adults ($n=49$) (where reported, ranging from $\ge 14$ to 21-64 years old), while some could be inferred based on the population description (i.e., veterans [68] or frequent users with alcohol problems [53]). Twenty-eight studies aimed to reduce ED visits in children, of which two assessed parents of newborns and two focused on school children (see Fig. 2). Twenty-seven studies focused on older adults, with definitions (where reported) ranging from $\ge 60$ years old to $\ge 75$ years old.

### Systematic reviews ($n=27$)

Table 2 presents the interventions evaluated across the SRs, as well as the populations targeted by the intervention studies within the SRs. Categories of interventions assessed included increasing primary care facilities, staff, and/or out-of hours care, implementing community health centres and care (e.g., health care workers in nursing homes, social work home visits, and home-based primary care), telephone triage and telemedicine advice, care strategies for older adults (e.g., end-of-life care by advanced nurses and using an assessment and treatment toolkit), barrier strategies (e.g, gatekeeping, payments or fees), education strategies, care programs (e.g., care plans, case management, and care coordination - as well as various combinations of these strategies), follow-up programs, and self-management strategies.

Ten of the SRs broadly defined their population inclusion criteria (i.e., they included adults or adults and children) [3, 7, 16, 204–206, 208, 209, 214, 217]. Of these, five examined specific interventions (i.e., walk-in centres or GP co-operatives [7], health literacy [206], transitional care strategies from hospital to home [214, 217], or multiple pre-defined interventions [16]). In the rest of this group of SRs, eligible interventions were not specified (e.g., ‘interventions to reduce ED visits’).

The remaining SRs targeted specific population groups, of which frequent ED users was the most common (6 SRs) [11–14, 211, 212]. Three of these SRs evaluated case management or care coordination strategies [11, 12, 212], while the other three included any type of intervention.

Three SRs included low-acuity patients presenting to the ED. One evaluated primary care interventions [8], one evaluated diversion strategies [10], and the third included any type of intervention that could reduce non-urgent paediatric ED visits [15].

Five SRs focused on older adults. Three concerned older patients with medical problems; one evaluated community-based care interventions [9], one examined transitional care programs [215], and one assessed home-based primary care [218]. Another evaluated any type of health service intervention for patients with community-dwelling people with dementia [207]. One targeted older adults in long-term care and evaluated on-site paramedic and other health treatment interventions [219]. Two SRs analysed children with medical conditions, one of which examined discharge process interventions [216] and the other evaluated ambulatory care programs [213].

Lastly, one SR assessed community-based care plans and care coordination for adults with at least two co-existing chronic medical conditions [210].

**Table 2:** Interventions evaluated, and population targeted in the systematic reviews

Interventions evaluated in the systematic reviews	Population targeted
**Capacity increase in non-ED settings**	
Walk-in centres	Adults (Crawford et al.) [7]; general population (Morley et al.) [3]; low acuity patients (Ismail et al.) [8]
Increasing the number of primary care centres or primary care physicians	General population (Flores-Mateo et al.) [204]
Capacity increase in non-ED settings (i.e., new clinics, expanded hours)	General population (Morgan et al.) [16]
GP-cooperatives (co-located or nearby EDs and mainly out-of-hours)	Adults (Crawford et al.) [7]; low-acuity patients (Ismail et al.) [8]
After hours GP	General population (Morley et al.) [3]
Out-of-hours medical services (by GP, commercially hired doctor, etc.)	General population (Flores-Mateo et al.) [204]
**Community health centres and care**	
Community health centres	Low-acuity patients (Ismail et al.) [8]
Community hospitals	Older patients with acute medical problems (Huntley et al.) [9]
Emergency nurse practitioner in residential care facilities	Low-acuity patients (Ismail et al.) [8]
Paramedic practitioners/emergency care practitioners in the community	Older patients with acute medical problems (Huntley et al.) [9]
Social work home visits to identify patient needs	Adult frequent ED users (Moe et al.) [14]
**Triage**	
Telephone triage and out-of-hours advice services	General population (Flores-Mateo et al.) [204]; low-acuity patients (Ismail et al.) [8]
Pre-hospital diversion (e.g., EMS or nurses/call centres offering alternative care such as PCPs or urgent care centres, or EMS ‘treat and release’)	Low-acuity patients (Morgan et al.) [16], (Raven et al.) [205]; patients seeking ED care (Kirkland et al.) [10], adult frequent ED users (Moe et al.) [14]
**Barrier strategies**	
Managed care (i.e., capitated payment of primary care physician or gatekeeping by PCP)	General population (Morgan et al.) [16]
Barrier interventions (i.e., out-of-pocket payments, flat fee, gatekeeping)	General population (Flores-Mateo et al.) [204]
**Patient financial incentives**	
Patient financial incentives (i.e., using costs to influence patients to use certain sites for care, or to use care efficiently)	General population (Morgan et al.) [16]
Financial penalties (i.e., copayments at ED)	Patients with Medicare or commercial insurance (Raven et al.) [205]
**Education strategies**	
Patient or parent education (e.g., on medical conditions and health care use)	General population (Flores-Mateo et al.) [204], (Morgan et al.); parents (Morrison et al.) [206]
Counselling on use of health care and the social system	Adult frequent ED users (Althaus et al.) [13]
Education (e.g., on primary care and preventative care, or appropriate PED use), information (e.g., on PCP office hours and other services), and support (e.g., follow-up and/or assisting to schedule a PCP appointment)	Children presenting to ED for non-urgent care (Poku and Hemingway) [15]
Educational materials/educational meetings (healthcare professional education)	Community-dwelling persons with dementia (Godard-Sebillotte et al.) [207]
**‘Social interventions’**	
‘Social interventions’ (i.e., public education campaign on proper use of the ED, financial disincentives, redirection, and provision of alternative clinics)	General population (Morley et al.) [3]
‘Social interventions’ (i.e., primary care referral, health education and counselling)	Frequent ED users (Hoot and Aronsky) [208]
**Care-coordination/program or management (not involving ED staff)**	
Care plans	Adults (Doshmangir et al.) [209]; adult frequent ED users (Moe et al.) [14]; adults with multimorbidity (Wasan et al.) [210]
Case management	Adults (Doshmangir et al.) [209]; adult frequent ED users (Althaus et al.) [13], (Di Mauro et al.) [11], (Iovan et al.) [211], (Kumar and Klein) [12], (Moe et al.) [14], (Raven et al.) [205]
Care coordination	Adult frequent ED users (Doshmangir et al.) [209], (Iovan et al.) [211]; adult frequent users of health care services (Tricco et al.) [212]; adults with multimorbidity (Wasan et al.) [210]
Medical care plan + one the following: social support, disease management or community health worker	Adult ED super-utilizers (Iovan et al.) [211]
Case management + one or more of the following: medical care plans or care coordination	Adult ED super-utilizers (Iovan et al.) [211]
Care coordination + (e.g., emergency care plans, medical plans, access to providers, expediated next day appointments, community health workers, telemedicine, etc.)	Adult frequent ED users (Althaus et al.) [13], (Iovan et al.); children with medical complexities (Pulcini et al.) [213]; patients with low acuity (Raven et al.) [205]
Transitional care programs (i.e., involving one or more of the following: care coordination, case management, care plans, education, health monitoring, management and intervention, exercise, physical and environmental assessments, medication support, self-management, etc.)	Adults, also including with COPD, heart failure and kidney transplant recipients (Chartrand et al.) [214], community-dwelling adults aged 60 or older with at least 1 medical diagnosis (Weeks et al.) [215]
One or more of the following strategies/features: case-management, shared care, teams, use of information and communication technology, and comprehensive geriatric assessment	Community-dwelling persons with dementia (Godard-Sebillotte et al.) [207]
**Follow-up**	
‘Discharge interventions’ (e.g., education with follow-up, self-management education and discharge game, post-discharge education sessions, needs assessments, home visits, telephone consultation, evaluation of home environment, etc.)	Paediatric patients (Auger et al.) [216]
Asthma education + primary care follow-up, care plan and home visit	Asthma patients (Raven et al.) [205]
**Self management**	
Self-management	Community-dwelling persons with dementia (Godard-Sebillotte et al.) [207]

## Discussion

This scoping review identified a large body of literature on interventions that aimed to reduce ED utilization. It did not aim to examine which interventions were most effective, but to map the evidence. In total, we identified 183 intervention studies (evaluating 187 interventions) and 27 systematic reviews. Across this literature, 15 broad intervention categories were identified and within these, several diverse strategies were assessed. An earlier scoping review of non-ED and ED interventions also reported some of the same interventions, but included fewer studies as they focused on those observed in the UK and France [220]. In our scoping review, the majority of primary studies (69%) were from the USA. In both the intervention studies and the SRs, case management, care coordination or multi-component interventions that incorporated one or more of these strategies, were assessed more often than any other type of strategy. A review of reviews also found coordination interventions to be the most common [221].

The highest proportion of primary studies were aimed at patients with specific medical conditions. For example, we found that 12 out of 13 studies examining patient education strategies targeted specific patient groups. A previous review has reported that this strategy has potential to reduce ED visits [16]. Although we have not evaluated effectiveness, and interventions aimed at specific population groups may not be transferable to broader groups, these interventions can inform new research ideas for other patient groups or the general patient population.

In the intervention studies, case management strategies were almost always tested on frequent users or high risk patients (with two exceptions), while different forms of care coordination were applied to broader population groups. Case management has been previously reported as an efficient way of reducing ED visits for frequent users [13]. Given that frequent visitors may make up between 21% and 28% of all ED visits [13], initiatives aimed at this population are important. However, the definition of frequent users widely varies in the literature [11–14], with $\ge 4$ visits per year appearing to be most common [222]. We have categorized frequent users and high risk patients separately using the authors’ definitions, although it appears that definitions for these two groups overlap. These findings emphasize the necessity for clearer and more uniform definitions.

Poor access to primary care has been identified as one of the causes of ED crowding in a previous review by Morley et al. [3]. In our scoping review, only seven out of the 27 (excluding studies examining communication) included studies located in primary care settings tested an intervention involving capacity increase, such as extended hours or opening of an extra clinic. Another scoping review [223] identified many studies involving capacity increase in primary care, but they included studies dating back to 1981. This indicated that fewer new studies are examining this type of intervention, and strategies to improve primary care access could be further explored as a potential solution to ED crowding. Notably, health care systems where primary care is well established tend to have fewer low acuity ED patients [224].

Increasing numbers of older patients has also been attributed to ED crowding [3]. Van den Heede’s review of reviews [221] included five reviews targeting older people, but most were ED based. We identified 28 intervention studies and five systematic reviews [9, 207, 215, 218, 219] that specifically evaluated interventions aimed at older adults. A previous systematic review found that 4% - 55% of acute transfers from a nursing home to ED’s are inappropriate [225], making it a potential target for interventions. Surprisingly, we found only eight intervention studies that were conducted in nursing homes, with six of them published in 2018 or earlier.

Despite the large amount of literature on this topic area, it is evident that certain areas receive more research attention, possibly driven by the high demands of specific patient groups visiting the ED. The interventions identified in our scoping review primarily focused on patient groups representing only a small proportion of the overall ED population. There is room for further research on resource-efficient, innovative strategies aimed at the general patient population and in low-acuity patients - and for more intervention studies addressing the needs of older patients and patients with mental health problems. To make it easier to identify studies on non-ED interventions to reduce ED utilization, we recommend that future reports adopt a more consistent use of terms like “ED/ER” combined with terms like “visits”, “presentations”, “use”, “utilization”, “crowding” or “overcrowding” in titles, abstracts and author keywords.

## Limitations

This scoping review has some limitations. We aimed to map a large topic area, and although the search strategy was broad with a large number of records retrieved, an even broader search strategy in more databases could have been employed. Some studies will therefore have been missed. In addition, we did not include any interventions that were conducted in the ED or used ED resources. This included interventions that were implemented outside the ED, but incorporated ED staff as part of the process. This was because we wanted to focus on ‘input’ solutions (e.g., see Morley et al. [3]). These interventions are important to consider, but other recent reviews specifically address these types of interventions (e.g., [4, 5]).

Some of the included primary studies did not clearly define their study population and/or intervention which made it difficult to categorize them accurately. Particularly, study authors often defined case management and care coordination in different ways, or did not describe the intervention enough to distinguish between them. Also, a number of studies did not report on who conducted the interventions, and this information would have been useful for analysis.

## Conclusions

We found a large number of primary and secondary studies investigating interventions to mitigate the influx of patients in the ED. A large proportion of these studies targeted patients with specific medical conditions, as well as frequent users and high-risk patients. The most commonly evaluated interventions were case management, care coordination, or other care strategies. Relatively fewer studies were conducted in patients with low acuity, older adults, and mental health patients. Further research may be needed in all three of these groups given their high prevalence in EDs.

### Supplementary information

Additional file 1. Details of the search strategies.

Additional file 2. Details of the included intervention studies.

Additional file 3. Details of the included systematic reviews.

## Data Availability

All data generated or analysed during this study are included in this published article [and its supplementary information files].
