# Supplementary material for: Non-emergency department (ED) interventions to reduce ED utilization: a scoping review
Source: BMC Emerg Med. 2024 Jul 12;24:117. doi: 10.1186/s12873-024-01028-4 (PMC11242019; doi:10.1186/s12873-024-01028-4)
Supplement: Supplementary file 1 — Additional file 1. Details of the search strategies. [file 12873_2024_1028_MOESM1_ESM.pdf]

Additional file 1: Details of the search strategies

MEDLINE

#	Searches
1	exp Emergency service hospital/
2	exp Emergency medical services/
3	(emergency adj2 (care or department? or unit? or center? or ward? or service?)).ti,kf.
4	(ED or EDs).ti,kf.
5	(ER or ERs).ti,kf.
6	(PER or PERs).ti,kf.
7	or/1-6 [Concept #1: ER]
8	exp Crowding/
9	((inappropriate* or appropriate* or unnecessary or "sub optimal" or optimal or "non urgent" or unurgent or avoidable) adj3 ("use" or "usage" or utilization or patient? or visit* or revisit? or attend* or admission? or readmission? or presentation?)).ti,ab,kf.
10	((non or "not" or low or "sub") adj3 (emergency or urgent or acute) adj3 ("use" or "usage" or utilization or patient? or visit* or revisit? or attend* or admission? or readmission?or presentation? or presentation?)).ti,ab,kf.
11	((high or frequent) adj2 (user? or visitor? or attend* or presentation?)).ti,ab,kf.
12	((patient? or visitor? or attend* or presentation?) adj2 (crowding* or overcrowd* or over-crowd* or saturat* or oversaturate* or over-saturate* or inflow or influx* or input? or flood* or overflood* or over-flood* or surge? or visit*)).ti,ab,kf.
13	overtriage.ti,ab,kf.
14	((ED? or ER?) adj3 (use* or utilization)).ti,ab,kf.
15	or/8-14 [Concept #2: ER visits]
16	exp Health Care Rationing/
17	exp Case Management/
18	exp Critical Pathways/
19	exp Decision Making/
20	exp Decision Support Systems, Clinical/
21	exp Health Services Misuse/
22	exp Patient Care Planning/
23	exp "Referral and Consultation"/
24	exp Remote Consultation/
25	exp Severity of Illness Index/
26	exp Trauma Severity Indices/
27	exp Health Services Research/
28	exp Cost-Effectiveness Analysis/
29	exp Triage/

30	((patient? or visit* or attend* or present*) adj3 (diversion or divert* or redirect* or bypass* or defer or deferring* or deferral? or deflect* or refer* or preven*)).ti,ab,kf.
31	((telemedicine or telephone or mobile or phone* or computer or app or virtual or digital) adj3 (screen* or triage or assess*)).ti,ab,kf.
32	((ER or ERs or ED or EDs) adj3 (predict* or anticipat* or forecast* or "machine learning" or "artificial intelligence" or AI or AIs)).ti,ab,kw.
33	or/16-32 [Concept #3: interventions to anticipate and reduce ER visits]
34	and/7,15,33 [Concepts #1-3 combined]
35	(comment or editorial or letter).pt.
36	34 not 35 [Exclude publication type filter]
37	limit 36 to yr="2008-current" [Publication date limit]

Embase

1	exp emergency ward/	Advanced
2	exp emergency care/	Advanced
3	exp emergency health service/	Advanced
4	exp emergency patient/	Advanced
5	exp emergency physician/	Advanced
6	exp emergency medical dispatcher/	Advanced
7	exp hospital emergency service/	Advanced
8	(emergency adj2 (care or department? or unit? or center? or ward? or service?)).ti,kw.	Advanced
9	(ED or EDs).ti,kw.	Advanced
10	(ER or ERs).ti,kw.	Advanced
11	(PER or PERs).ti,kw.	Advanced
12	or/1-11 [Concept #1: ER]	Advanced
13	exp Crowding/	Advanced
14	((inappropriate* or appropriate* or unnecessary or "sub optimal" or optimal or "non urgent" or unurgent or avoidable) adj3 ("use" or "usage" or utilization or patient? or visit* or revisit? or attend* or admission? or readmission? or presentation?)).ti,ab,kw.	Advanced
15	((non or "not" or low or "sub") adj3 (emergency or urgent or acute) adj3 ("use" or "usage" or utilization or patient? or visit* or revisit? or attend* or admission? or readmission? or presentation?)).ti,ab,kw.	Advanced
16	((high or frequent) adj2 (user? or visitor? or attend* or presentation?)).ti,ab,kw.	Advanced
17	((patient? or visitor? or attend*) adj2 (crowding* or overcrowd* or over-crowd* or saturat* or oversaturate* or over-saturate* or inflow or influx* or input? or flood* or	Advanced

	overflood* or over-flood* or surge? or visit* or presentation?)).ti,ab,kw.	
18	overtriage.ti,ab,kw.	Advanced
19	((ED? or ER?) adj3 (use* or utilization)).ti,ab,kw.	Advanced
20	or/13-19 [Concept #2: ER Attendance]	Advanced
21	*health care organization/	Advanced
22	exp health care planning/	Advanced
23	exp resource allocation/	Advanced
24	exp health care utilization/	Advanced
25	exp clinical pathway/	Advanced
26	exp decision making/	Advanced
27	exp decision support system/	Advanced
28	exp patient care planning/	Advanced
67	exp patient referral/	Advanced
29	exp teleconsultation/	Advanced
30	exp "severity of illness index"/	Advanced
31	exp injury scale/	Advanced
32	exp "utilization review"/	Advanced
33	exp health services research/	Advanced
34	exp "cost effectiveness analysis"/	Advanced
35	((patient? or visit* or attend* or present*) adj3 (diversion or divert* or redirect* or bypass* or defer or deferring* or deferral? or deflect* or refer* or preven*)).ti,ab,kw.	Advanced
36	((telemedicine or telephone or mobile or phone* or computer or app or virtual or digital) adj3 (screen* or triage or assess*)).ti,ab,kw.	Advanced
37	((ER or ERs or ED or EDs) adj3 (predict* or anticipat* or forecast* or "machine learning" or "artificial intelligence" or AI or AIs)).ti,ab,kw.	Advanced
38	or/21-37[Concept #3: interventions to anticipate and reduce ER visits]	Advanced
39	and/12,20,38 [Concepts #1-3 combined]	Advanced
40	(comment or editorial or letter).pt.	Advanced
41	39 not 40 [editorial or letter records removed]	Advanced
42	limit 41 to yr="2008-current" [Publication date limit]	Advanced
