# Supplementary material for: Non-emergency department (ED) interventions to reduce ED utilization: a scoping review
Source: BMC Emerg Med. 2024 Jul 12;24:117. doi: 10.1186/s12873-024-01028-4 (PMC11242019; doi:10.1186/s12873-024-01028-4)
Supplement: Supplementary file 2 — Additional file 2. Details of the included intervention studies. [file 12873_2024_1028_MOESM2_ESM.pdf]

Additional file 2: Details of the included intervention studies

Author, year	Study design	Patient population as reported in the paper	Intervention as reported in the paper	Intervention category
Abdur-Rahman et al., 2016	Before-and-after	Patients seen in an academic urban and children's hospital EDs from July of 2013 to October of 2014 with asthma exacerbation who were referred to and completed Community Asthma Programs (CAP)	Community Asthma Programs (CAP)	Care program
Adam et al., 2010	Controlled before-and-after	Frequent Attenders in a Family Medicine residency clinic	Interdisciplinary Care Team: During 1 hr each week, the Care Team met to review the health care status of a case patient. The patient's primary physician summarized the patient's medical, mental, and social health, including positive and negative care experiences. The Care Team asked questions, made observations, and suggested solutions. At the close of the meeting, the primary physician summarized options for improved care and recorded them in a care plan.	Care coordination
Adesera et al., 2011	Before-and-after	Patients presenting to a family medical center (FMC)	Posters encouraging patients to contact the family medical centre (FMC) before going to the ED were placed in the waiting area, each exam room, and the triage room in the FMC. Patients seen in the ED for non-emergent reasons aged 18 to 70 years old recieved a letter encouraging them to visit the FMC firsts.	Education of patients
Akiya et al., 2021	Non-randomized controlled trial	Older adults who were (1) overusing ED or hospital-based medical care; or (2) struggling with a nonclinical issue that affected their health (eg, transportation or food insecurity)	Community Care Connections (CCC) program, a cross-sector collaboration designed to align social and health care services for older adults. The CCC program provides intensive case management and health care coordination to older adults. The program embedded social work care managers in physician offices and established referral relationships with other local medical providers.	Care program
Ali et al., 2019	Before-and-after	All patients with asthma in our pulmonary clinic who participated in structured group education. Only those patients who were followed for one year before and one year after asthma education were included.	Standardized group asthma education for 8–12 patients was offered in the pulmonary clinic by certified asthma educators, including bilingual (English and Spanish) respiratory therapist, clinical pharmacist, and pulmonologist. The two education sessions consisted of meetings for two to two and a half consecutive hours per week. The standardized education tools used were based on the GINA guidelines and included a PowerPoint presentation, education booklets, a lung model, spacers, peak flow meters, and placebo inhalers for demonstration purposes. Both weekly sessions were held by the same educators.	Education of patients
Allen et al., 2016	Before-and-after	Students from 129 schools	School Based Asthma Therapy (SBAT). Controller therapy is adjusted post discussion with the primary asthma provider, insurance authorization is obtained, inhalers are provided to home and school, then doses are administered at least daily at school. If follow-up evaluation susing Asthma Control Test (ACT) scores identify a child in on going poor control, asthma providers are alerted and medications adjusted.	School based therapy
Alshabani et al., 2018	Before-and-after	COPD patients with history of increased healthcare utilization	Electronic inhaler monitoring (EIM) consisting of tools that allow real-time tracking of medication utilization. EIM alerted the monitoring team when patients did not use maintenance inhalers for 4 consecutive days and when rescue inhaler use increased compared to baseline. Patients were contacted in response to these alerts to encourage adherence and assess for presence of a COPD exacerbation. Adherence data were made available to treating physicians.	Medication management

Anantharaman et al., 2008	Retrospective cohort	1. General population thorugh media<br>2. Patients visiting an ED<br>3. Non-emergency patients visiting an public EDs<br>4. Re-directed patients from an ED and those with minor complaints	1. Public education campaigns on the proper use of EDs<br>2. Financial disincentives directed at patients attending EDs<br>3. Redirection of non-emergency patients from public EDs<br>4. Provision of alternative clinics for re-directed patients and those with minor complaints	1. Public education<br>2. Patient financial incentives<br>3. Redirection after triage<br>4. Additional clinics
Anugu et al., 2017	Prospective cohort	Pediatric patients (newborns through those aged <18 years) at 5 pediatric primary care offices in Suffolk County, New York, considered by a clinician to be "at risk" for poor health outcomes	Home visits from trained Community Health Workers (CHWs) to support adherence to recommended care. A monthly visit for 45- to 60-minutes. CHWs may help families keep track of a child's medical information, appointments, insurance information, and medications; communicate with primary medical physician and specialists; or troubleshoot issues between office visits (eg, transportation, medication refills). After each visit, summary notes were given to clinicians for review.	Home visit
Apter et al., 2020	RCT	Adults with moderate to severe asthma from clinics serving low-income neighborhoods	A patient advocate who coached, modeled, and assisted participants with preparations for asthma-related medical visits, attended visits, and confirmed participants' understanding of providers' recommendations.	Care coordination
Arain et al., 2013	Time series	Patients with minor cases (GP-type cases) were defined as those patients who were referred or discharged from the ED without needing any investigation, or only some low-cost investigations as defined by the tariff codes used by the primary care trust (PCT).	GP led walk-in centres (WICs). The centre provides walk-in services from 8:00 to 21:00, 7 days a week.	Additional clinics
Araujo et al., 2020	RCT	Patients with metastatic cancer	Patient navigation (PN). Patients in the PN arm received supportive care (SC) interventions by a navigator-led multidisciplinary team (palliative care, physical therapy, geriatrics, psychology) in the first 12 weeks after diagnosis.	Care coordination
Arbour et al., 2024	Before-and-after	Patients with housing-related needs that extend beyond homelessness	Primary Care–Based Housing Program. The team includes housing advocates, community resource specialists, community health workers, and a medical-legal partner that provides legal information and problem-solving education at biweekly case reviews. Housing advocates support patients experiencing homelessness or unstable or unsafe housing by assisting them with shelter placement, landlord or property management negotiation, reasonable accommodation requests, and housing application assistance, among other services.	Care coordination and housing
Arendts et al., 2018	Cluster RCT	People aged 65 years and older living in one of six residental aged care facilities (RACF)	Nurse practitioner that worked with general practitioners (primary care). The nurses used a best practice resource folder and were responsible for the care processes being delivered. The best practice resource folder contained guidelines for comprehensive medical assessment; patient +/− family education regarding diagnosis and prognosis; care pathways for specific acute illnesses, palliative care plan for management of current and anticipated future symptoms; advance care planning; medication review; and a review of unplanned hospitalisations at regular meetings utilising root cause analysis.	Care coordination
Arora et al., 2013	RCT	Patients 18 years or older with poorly controlled type 2 diabetes (Hb A1C level greater than or equal to 8%) in the ED at Los Angeles County Hospital	In the TExT-MED system, patients received 2 messages (9 am and 5 pm) delivered to their mobile telephones daily for 6 months.<br>Educational/motivational (1 per day), medication reminders (3 per week), healthy living challenges (2 per week), trivia (2 per week).	Self-management

Arrowsmith et al., 2019	Before-and-after	Patients in a single large community oncology practice where more than 3,200 cancer patients were treated during 2016	Several low-cost care coordination (CC) interventions: Initiated after hours call process with access to EMR and patient access to bidirectional real-time messaging with care team members; Implemented new in-office process to “close the loop” on patient evaluations by creating follow up guidelines for symptomatic telephone triage and in-clinic patient evaluations; Implemented a standard 48 hour follow up process for all ED visits (EDV) and hospital admissions; Increased patient awareness of telephone triage services during and after clinic hours by: augmenting new patient education by staff, developing a magnetic reminder to call the office for non-emergent and emergency situations, and instructions for use of after hours call system.	Care coordination
Ascher et al., 2019	Before-and-after	High-risk patients with patterns of high utilization	Community Paramedicine Visit Program (CPVP): Met with clinic staff, management and PCPs to create workflow.	Community paramedic program
Augustine et al., 2020	Before-and-after	Patients seen at primary care clinics at VA Medical Centers (VAMC)	Extended hours at 101 primary care clinics over a 15-month period from July 2017 to September 2018.	Increased primary care hours
Beal et al., 2015	Before-and-after	Patients younger than 21 years with "eczema", "dermatitis" and "atopic dermatitis" seen in the Cardinal Glennon Children's Medical Center (CGCMC) ED	Implementing a full-time pediatric dermatology service	Additional services
Bell et al., 2016	RCT	Patients aged 18 and older that were hospitalized for acute coronary syndromes (ACS) and/or acute decompensated heart failure (ADHF) in two academic medical centers; Vanderbilt University Hospital (VUH) in Nashville, Tennessee, and Brigham and Women’s Hospital (BWH) in Boston, Massachusetts.	Four components: 1. Pharmacists reconciled preadmission medications and discharge medications with the patient and reported any inconsistencies to the medical team, prior to hospital discharge. 2. The pharmacist provided tailored counseling, which included assessing patient understanding of the medication regimen, barriers to medication adherence, and trouble-shooting barriers while the patient was in the hospital. 3. At discharge, the pharmacist provided additional counseling, an illustrated medication schedule showing the discharge regimen, and a pillbox, which the patient practiced filling. The pharmacist also employed a teach-back technique to ensure patient understanding. 4. Within four days after hospital discharge, study coordinators contacted the patients and inquired about general health, symptoms, and any medication-related problems such as regimen confusion, non-adherence, or side effects.	Pharmacist involvement
Belzile et al., 2023	Retrospective controlled cohort	All outpatients who tested positive for COVID-19	Outpatients (COVIDEO) programme involving virtual assessments. The COVIDEO team contacted the patient by telephone or video platform, to provide a structured assessment. Follow-up virtual visits were scheduled regularly, with more frequent visits arranged for patients identified as high risk of severe disease; in the first wave all patients were followed until the end of their isolation period, but in subsequent waves routine follow-up was only arranged for higher risk patients.	Telephone follow-up and telemonitoring
Bessert et al., 2023	Before-and-after	Adult patients who seek to walk-in to the ED	An urgent care walk-in clinic (WIC) was opened in the immediate vicinity of the ED. This enabled the provision of general medical care to low- urgency patients between 4 pm and midnight. Since then, it has been possible to refer patients there from the ED registration desk during WIC operating hours without the patients being admitted to the ED.	Additional clinics
Bhutta and Chrusciel, 2021	Before-and-after	ED patients with epilepsy	Quality improvement metrics that included laminated cards with personalized seizure action plans and an EMR template for documentation of those plans.	Self-management
Biesboer et al., 2024	RCT	Adult (≥18 years old) patients admitted to the trauma surgery service at an urban, Midwest adult Level 1 trauma center with a firearm-related injury	Post Discharge Care Team (PDCT) consisting of a dedicated trauma nurse navigator and medical social worker. The PDCT nurse provided education and set expectations regarding injuries, wound care, and outpatient follow up. The PDCT social worker performed a comprehensive assessment to identify concerns including housing and financial instability, food insecurity, or transportation issues.	Follow-up program

Biese et al., 2017	RCT	Individuals aged 65 and older discharged from the ED without hospital admission from a large academic medical center	A scripted 30-day follow-up telephone interview by a nurse. The questionnaire aimed to identify potential problems with their care transition and to offer advice on medication reconciliation and procurement, review of instructions and procurement of supplies for nonmedicinal treatments, review and re-emphasis of postdischarge instructions, reinforcement of follow-up appointments and assistance in making appointments, and current state of health and advice if not feeling well. Participants were asked about visits to any ED within the 30 days since their initial ED or hospital visit, visits to their PCP or specialists.	Telephone follow-up
Blecker et al., 2020	Controlled before and after	Patients with complex diabetes age >= 18 years (1) type 1 diabetes; (2) type 2 diabetes on insulin; (3) type 2 diabetes with a glycated hemoglobin (HbA1c) > 9% (75mmol/mol). Recruted from clinics that serves underserved populations.	The intervention utilized a tele-mentoring model to connect primary care providers and community health workers (who assist with diabetes management) with specialists for training in diabetes care (this was done using Endocrinology ECHO model which is an intervention involving education in complex disease management).	Education of staff and case management
Block et al., 2013	Time series	Uninsured and underinsured patients referred from East Baltimore Medical Center (EBMC) and who were evaluated for medical necessity to join The Access Partnership (TAP)	TAP-covered services included all specialty care available at the academic medical center. Subsequent visits, diagnostics, and hospitalizations stemming from the referral were covered by the program as well. The Access Partnership navigators also facilitated engagement in primary care. The navigator communicated with the primary care physician about the status of the referral and arranged timely follow-up with the primary care physician after the specialty visit.	Care coordination
Bodenmann et al., 2017	RCT	Frequent ED users at least 18 years of age. Those who made five or more ED visits during the prior 12 months, including the index visit.	The case managment (CM) team provided individualized services to each participant in the intervention group, emphasizing care coordination and facilitating communication between health care team members. Specifically, CM team members provided counseling, based on motivational interviewing and cross-cultural competences, on substance abuse (if applicable) and use of medical services. Intervention group received the CM intervention at baseline and at 1, 3, and 5 months.	Case management
Borde et al., 2023	Before-and-after	ED super-utilizers	An integrated practice unit (IPU) that provides a multidisciplinary approach to patient care, typically involving a primary care provider, registered nurse, social worker, and pharmacist	Additional clinics
Bradley et al., 2018	RCT	Subjects who had household incomes below 100% of the federal poverty level, had no other health insurance coverage. Childless adults, many of whom are likely to have multiple chronic conditions. No prior VCC (Virginia Coordinated care) coverage in the past 12 months or a VCC re-enrollee with no PCP or specialist visit in the prior 9 months; aged 21–64 years; spoke English; and resided in the community (e.g., not homeless or living at a drug or alcohol rehabilitation facility).	Subjects were randomly assigned and given six months to see their primary care provider. If we received a health care claim indicating that the subject saw their PCP within this six-month period, we paid the subject either $25 or $50 depending on group assignment. Subjects were paid only once and only for the first PCP visit. Subjects in the $0 group did not receive a payment other than the initial $10 to complete the baseline survey.	Patient financial incentives
Brown et al., 2022	RCT	High-risk patients from the population of Medicaid beneficiaries. Adult aged 18 years or older residing in Contra Costa County	Case management services designed to address patients' social and health needs. Case managers took the following 3 steps: 1) social needs screening to inquire about patients' needs, including food, transportation, housing, finances, employment,legal support, safety, and social support, as well as unmet medical, behavioral health, dental, and vision care needs; 2) worked collaboratively to develop a patient-centered care plan; and 3) supported patients' progress on goals through coaching, help with applications for public benefits, referrals to social services, assistance communicating with health care providers and social service agencies, and direct access to resources managed by CommunityConnect (cell phones, emergency housing funds, and legal aid).	Case management

Brown et al., 2020	RCT	Participant eligibility was based on frequent or avoidable acute care service us (emergency department [ED] visits and hospitalizations), the presence of uncontrolled chronic conditions, and provider referral	The Care Connections Program (CCP) embedded community health workers (CHWs) in 8 primary care clinics in the Los Angeles County Department of Health Services (LAC DHS). CHWs guided several patient interventions for clinically and socially complex patients, including medication review, health system navigation, home visits, health coaching, care transitions support, social support, and linkages to appropriate resources.	Care coordination
Bruzzese et al., 2011	RCT	9th and 10th graders with moderate to severe persistent asthma	ASMA consists of two complementary components: (1) an 8-week intensive program for the students, and (2) academic detailing for the adolescents' medical providers. The student intervention consists of three 45- to 60-minute group sessions, and individual tailored coaching sessions held at least once per week for 5 weeks. Sessions are delivered by trained health educators during the school day.	School based therapy
Buckley et al., 2010	Time series	Low-urgency patients seen in a after-hours general practice clinic	Opening of the after-hours clinic, of patients triaged as Australasian Triage Scale (ATS) category 4 or 5 (at any time of day, and during the hours of operation of the clinic), and of patients triaged as ATS category 1, 2 or 3 (at any time of day).	Additional clinics
Burnett et al., 2023	Before-and-after	Patients discharged from an acute admission with a diagnosis of congestive heart failure (CHF), acute myocardial infarction (AMI) and chronic obstructive pulmonary disease (COPD)	Upon discharge the community paramedic (CP) visited the patient in their home within 48 hours and subsequently 1–2 times per week for 30 days. While the home visit contained standardized elements, the CPs were allowed to individualize how they prioritized the specific required elements based on their needs assessment for each patient.	Home visit
Calvo et al., 2015	RCT follow-up (2 years after)	Adolescents with early-onset psychosis and their families	PE (Psychoeducational) group: The initiation phase consisted of 3 individual sessions of 50 minutes each, conducted for families and adolescents separately. The group phase consisted of 2 separate group interventions of 12 biweekly, 90-minute, structured sessions, 1 for patients and the other for parents. Groups focused specifically on problem-solving strategies to manage difficulties with daily living associated with the disease to mitigate crises and to prevent relapses. Patients and their families received written PE material within this treatment modality. The NS (nonstructured) also had an initiation phase (3 individual sessions for parents and adolescents) and 12 biweekly support group sessions. No written material was provided in this modality.	Education of patients and parents
Campos-Gomez and Campos-Gomez, 2022	Retrospective cohort	Lung cancer patients	Preventive care intervention: Patients and their primary caregivers in preventive care intervention were instructed, during clinical visits, on how to better manage the side effects of the treatment at home including care coordination strategies during outpatient systemic therapy treatment.	Prevention program
Canabal Sanmartin et al., 2012	Before-and-after	Non-controlled asthmatic patients (aged between 4 and 62 years), treated in an allergy unit	The intervention consisted of adjustment of the treatment according to the severity of the disease, followed by an educational practical programme that included: understanding the difference between 'controller' and 'reliever' medication, explaining the correct use of the inhalers, recognizing the signs of an exacerbation and the use of appropriate medication, monitoring of the patients' status by assessing the symptoms and PEF measurements, and avoiding of trigger factors. All the patients received a written plan of action in case of asthma symptoms worsening and exacerbations.	Education of patients
Carey et al., 2017	Retrospective cohort	Adults (aged 18-84) with intellectual disability (ID)	Annual health checks	Health checks
Chan et al., 2017	Before-and-after	Children under 12 months visiting academic primary care clinic serving predominantly low-income families	Interventions focused on expanding urgent care (UC) and nursing-line access, improving parents’ awareness of these ED-alternatives, and enhancing caregiver knowledge of infant care.	Increased clinic access and education of parents

Chang et al., 2021	RCT	High-risk veterans with recent hospitalization or ED visit	Primary care Intensive Management teams (PIM). The PIM teams were led by primary care physicians and generally consisted of nurses, social workers, and psychologists. Three teams also included peer support specialists. The teams performed comprehensive assessments, preventative home visits, transitional care management, medication management, care coordination, health coaching, patient and caregiver education, case management for social needs, and advance care planning.	Care program
Chapman et al., 2019	Before-and-after	Frail, older adults with multiple long-term conditions	Okay to Stay is a simple plan for people with long-term conditions to help them remain in their own home if they suffer an acute exacerbation in their health. Once a plan has been formulated in close collaboration with the patient and relatives/carers, each patient is supported with regular visits by community matrons or other members othe integrated care team, in combination with weekly phone calls (if appropriate) to monitor and manage their health conditions. Following initiation of the plan, should the patient become unwell and access urgent care, such as the out of hours GP or the ambulance service, the responding clinician can then access the Okay to Stay plan as part of their care provision.	Care plan
Chaudhuri et al., 2019	Before-and-after	Patients with cancer-related symptoms	An acute care clinic (ACC), appropriately staffed to provide ancillary services, was set up in an academic hospital outpatient setting with hours of operations 10:30-8:00 pm to effectively manage patients with cancer-related symptoms.	Additional clinics
Chen et al., 2014	RCT	Patients diagnosed with lung cancer	Patient-centered home-telemonitoring in cancer self-management	Telemonitoring and self-management
Chin et al., 2022	Before-and-after	Low-acuity patients	In this progam, a patient who attended a GPFirst clinic and then subsequently referred to the ED will qualify for an ED attendance fee discount.	Patient financial incentives
Choi et al., 2022	Retrospective cohort	Patients with Severe Mental Illness (SMI) And Cardiovascular Disease (CVD)	Medicine in Psychiatry (MIPS) is a SMI-focused primary care clinic co-located alongside community mental health services. MIPS is a form of “reverse integration” that allows for warm-handoffs and clinical collaboration between primary care and mental health providers to improve access to comprehensive care.	Co-location of clinics
Chow et al., 2019	Before-and-after	Patients referred to GERIPACT by primary care providers. Complex elderly patients at high-risk for incurring expensive health care system use (ie. frequent emergency room visits or hospitalizations).	GERIPACT is an inter-professional team of 2 clinicians, 1 social worker, and 1 care coordinator. It's services include frequent office visits for medical and social work needs, frequent telephone contact to patient and caregivers, home visits, specialty visit accompaniment, and a 24/7 telephone hotline.	Care program
Colligan et al., 2017	Retrospective cohort	Medicare fee-for-service patients with active cancer (breast, lung, lymphoma, and colorectal cancers).	Two interventions were evaluated in two different patients groups: COME HOME (Community Oncology Medical home) and PCCP (Patient Care Connect Program) practices).<br><br>1. COME HOME: The model was implemented at 7 outpatient oncology practices. Triage pathways to help first responders and nurses identify and manage patient symptoms; extended access to outpatient care during evenings and weekends for the practices' symptomatic patients after chemotherapy, radiation therapy, or surgery; and diagnosis and treatment pathways based on nationally recognized, evidence-based standards to guide clinical decision making and support patient self-management.<br><br>2. PCCP: A patient navigation program at 12 oncology practices. The PCCP employed and trained nonclinical navigators who educated and enpowered cancer patients and survivors, connected patients and caregivers with resources, and improved adherence to care plans.	1. Care model<br>2. Care coordination

Connolly et al., 2018	Before-and-after	21 long-term care facilities with above-average rates of hospital presentations during the three-month period excluding the calendar month prior to intervention start. All facility residents during the study period were included.	Aged Residential Care Intervention Project (ARCHIP): (a) baseline facility assessment identifying needs, and facility care plan developed by study gerontology nurse (GNS) specialist and facility senior nurse; (b) clinical coaching for long-term care (LTC) nurses & care-givers, withincreased clinical coaching time at each facility; (c) three 1-hour multidisciplinary team meetings, including medication review, by study geriatrician, GNS, pharmacist & facility general practitioner and senior LTC nurse(s).	Care program
Crane et al., 2012	Controlled before-and-after	Uninsured, frequent users of the emergency department (at least 6 times during a 12-month period), with a family income of 200% or below the federal poverty guidelines.	The program was based at the Free Clinics of Henderson County. It included 4 components: twice-weekly drop-in group medical appointments staffed by an interdisciplinary team of a family physician, behavioral health professional, and nurse case manager; direct telephone access to registred nurse care manager; small group "life skills and support" sessions with the care manager; short, individual sessions after the group medical visit.	Care program
Curfman et al., 2021	Before-and-after	Children with medical complexity	vKids at Home is a virtual service designed to improvequality of life for children with medical complexity. Patients are provided with an app or iPad and peripheral devices for transmitting vital signs. Patients complete an intake process with a comprehensive assessment of their medical history, psychosocial history, equipment, medications, and providers involved in their care. Clinical action plans are veried with all involved care team members and vKids continually communicates with the patients' careteams to escalate to in-person visits when necessary. Patients complete a daily, proactive digital touchpoint with the ability to escalate to text message, phone, or video with the virtual care team of nurses, nursepractitioners, social workers, and physicians.	Telemonitoring
Davis et al., 2018	Before-and-after	Parents of pediatric patients seen in the family medicine residency clinic at New Hanover Regional Medical Center	To improve parent education, posters describing our walk-in clinic were placed in the waiting area as well as examination rooms, office staff and physicians were given updated scripting to discuss with patients, the after-hours phone triage line was updated, and bookmarks that described our walk-in clinic as well as an after-hours call line were placed in the books our children get at each well-child examination.	Education of parents and clinic access
de Jong et al., 2017	RCT	Patients between 18 and 75 years of age, fulfilling the international diagnostic criteria for inflammatory bowel disease	Care via a telemedicine system (MyIBDcoach). This system includes monthly monitoring modules, which contain questions regarding disease activity, medication use, treatment adherence, treatment satisfaction, and side-effects, including infections. The system also includes questions on factors affecting disease (including nutritional status, smoking, stress, life events, anxiety and depression, social support, physical exercise, and self-management skills), and patient-reported outcome measures on quality of life and work productivity. It allows the patient to communicate with health care workers and gives feedback to the back office and the patient.	Telemonitoring and self-management
DeCamp et al., 2020	RCT	Parents or legal guardians of publicly insured, singleton US-born infants <2 months of age at a academic general pediatrics clinic. Minimum parent age of 18 years, self-identification as Latino or Latina.	The Salud al Día intervention, consisted of interactive text messages throughout the child's first year of life and an educational video: appointment reminders, support for obtaining medicines, support for completing referrals, and illness care monitoring and education.	Education of parents and support
DeVries et al., 2013	Controlled before-and-after	Commercially insured members from the employer group where ERUMI was implemented	Emergency Room Utilization Management Initiative (ERUMI): an increase in the copayment for ED visits from $100 to $200 and an educational brochure mailed to each household. Members with nonurgent ED visits received a reinforcement mailing and an automated follow-up call regarding ED alternatives they could have chosen.	Patient financial incentives
Diamant et al., 2011	Before-and-after	Homeless, frequent ED user with a physical disability or chronic illness	Access to Housing for Health (AHH) intervention which focuses on: 1) use of medical services, 2) enrollment into benefits and community services, 3) individual health status, and 4) stability in housing.	Care coordination and housing

Dolton and Pathania, 2016	Controlled before-and-after	all patients visiting the Accident & Emergency (A&E) units of hospitals	7-day opening of GP practices	Increased primary care hours
Dukelow et al., 2019	Before-and-after	Individuals who received a new or increased service from Community Care Access Centre (CCAC) Older patients living at home who might benefit from home care services	The Community Referral by Emergency Medical Services (CREMS) program. The program allows paramedics interacting with a patient to directly refer those in need of home care support to their local Community Care Access Centre (CCAC) for needs assessment. If indicated, subsequent referrals are made to specific services (e.g. nursing, physiotherapy and geriatrics) by CCAC.	EMS care coordination
Echeverry et al., 2015	Before-and-after	Patients diagnosed with Class III or IV heart failure	An Adult-Gerontological nurse practitioner (NP), made home visits to patients on a monthly and as-needed basis. Once the patient was discharged home from a hospital visit, the NP scheduled a home visit within 7 to 10 days to ensure the patient had the proper instructions on medication administration, was able to obtain medications, and had the appropriate referrals in place.	Home visit
Edelman et al., 2010	RCT	Patients receiving primary care at the Durham or Richmond VAMC with poorly controlled diabetes (A1c>=7.5%) and blood pressure (BP) (systolic BP >= 140 or diastolic BP>=90)	Groups of the same 7-8 patients met with the same pharmacist and general internist each visit; there were different physicians and pharmacistsacross groups. Each session included group education and structured group interactions moderated by a registered nurse or certified diabetes educator. Additionally, individual medication adjustments were made by the pharmacist and physician to manage A1c and BP. Each group met every two months for a year.	Education of patients
Eicken et al., 2020	Before-and-after	Adult patients with diabetes	The innovative community paramedicine program (CPP) provided in-home disease management, education, and care/community resource connection by paramedics.	Community paramedic program
ElBestawi et al., 2018	Prospective cohort	Long-Term Care (LTC) residents	PREVIEW-ED. The new tool, Practical Routine Elder Variants Indicate Early Warning for Emergency Department (PREVIEW-ED), was designed with nine indicators, each with weighted signs and symptoms depending on the severity. The tool design prompted PSWs (Personal Support Workers) to indicate whether the resident they were caring for seemed to be “normal” for that resident or whether there was a change.	Screening tool
Elston et al., 2022	Controlled before-and-after	Patients aged 18 years or older, who were predicted to be at high risk of attending ED within the next 6 months were defined as frequent attenders.	Intervention recipients were assigned a health navigator coach typically contacting them every 1–2 weeks by telephone and over a period of 6 to 9 months, during which they worked with the patient (using standardised templates) to optimise their medical treatment, nursing and care coordination, motivated them to improve their self-care and well-being (developing a joint plan with goal-setting).	Care coordination
Epperson and Shipman, 2019	Before-and-after	A selected group of the highest utilizers involved in community care coordination (CCC) program	Community care coordination (CCC). The CCC team consisted of two registered nurses, two social workers, a nurse practitioner, and two bilingual community health workers who followed patients up to 6 months post-discharge from the ED or hospital. The team made frequent visits to patient homes to assess and assist with social and health care needs. They made frequent face-to-face contact with super-utilizers to coordinate individual care plans, establish follow-up, and educate home-management for chronic conditions to addressed underlying social barriers and provided necessary resources to prevent disease progression necessitating acute care services.	Care coordination
Ernecoff et al., 2021	Retrospective case-control	People with serious or complex chronic illnesses	The Advanced Illness Care (AIC) Program is a home-based, nurse practitioner-led wraparound service that includes social work and case management, tailored to support patients and families as they face the burdens of navigating serious illness.	Care coordination
Eustache et al., 2023	Prospective cohort	Patients who had elective abdominopelvic colorectal surgery	A mobile phone app that comprised of patient education material, daily questionnaires assessing postdischarge recovery, and patient-provider chat function was used.	Self-management

Farnham et al., 2017	Retrospective cohort	Children with bronchiolitis, gastroenteritis, fever with a cause, croup, viral-induced wheeze or exacerbation of asthma	The H@H (Hospital at Home) team provides care fo children in their home instead of the traditional hospital setting. The team comprises one clinical band 7 and four senior band 6 nurses. It involves relocating treamtent for selected conditions to the home environment, promoting parents' and carers' confidence so they can manage unwell children safely at home, and provide health education, support and advice.	Home visit
Farrelly et al., 2023	Before-and-after	Nursing home residents	A framework to deliver specialist acute geriatric care to 4 Residential Care Facilities. Direct access by phone to a daily team lead was developed and provided on a Monday-Friday 8 am -5 pm basis. Referrals were triaged and acute response service provided through ANP and consultant geriatrician. Protocols to support iv fluid and antibiotic administration were agreed. Framework RCFs also engaged in specific Quality Improvement (QI) initiatives that focussed on falls reduction, skin integrity assessment, appropriate use of ED and safe staffing.	Care coordination and triage
Fieldstone et al., 2013	Before-and-after	Caregivers of children aged 7 months to 5 years recruited from primary care centers	Didactic session and skills demonstration, following a highly participatory curriculum developed with adult learning principles in mind. It was taught by trained pediatric nurses from the Children's Hospital ED and addressed fever, colds, and minor trauma - the most common nonurgent pediatric chief complaints presenting to EDs.	Education of parents
Foucaud et al., 2022	Before-and-after	Patients aged 75 or above, and were made by the patient him/herself, his/her family, a health professional (GP, nurse, first aid, home care professional), or following the activation of a medical alarm system for older persons; between 8 am and 6 pm on weekdays (Monday to Friday), in order to correspond to open hours when GP and geriatric services are available.	The REGESA intervention consisted in providing specific training and tools to dispatching physicians to improve their knowledge of the specific aspects of emergency care in older patients and to help them better manage older patients dispatching, notably by gathering specific key information and by using other pathways than the ED when possible.	Education of staff
Frail et al., 2012	Cluster RCT	Medicare home health population	Telephonic medication therapy management (MTM) service: 1) initial phone call by a pharmacy technician to verify an active medication list, 2) pharmacist-completed medication therapy review by telephone, and 3) follow-up pharmacist phone calls at days 7 and 30. Patients also received a medication action plan and personal medication record. Pharmacists intervened with prescribers and patients/caregivers to resolve identified drug therapy problems.	Telephone follow-up with pharmacist
Gajra et al., 2023	Before-and-after	Patients in Oncology Care Model (OCM) practice, the Center for Cancer and Blood Disorders practice.	Jvion Care Optimization and Recommendation Enhancement augmented intelligence (AI) tool, which applied continuous machine learning (ML) to predict risk of preventable harm (avoidable ACU) and generated patient-specific recommendations. At their discretion, nurses contacted at-risk patients with interventions to avert the ACU.	Screening tool with AI
García-Gollarte et al., 2014	Cluster RCT	Persons older than 65 years, who had been living in the nursing home for at least 3 months	10 hours educational program for physicians, followed by on demand support by phone (nursing home staff, physician and nurses). A nursing home physician, expert in drug use in older people, delivered a structured educational intervention. The program included general aspects of prescription and drug use in geriatric patients, how to reduce the number of drugs, to perform a regular review of medications, to avoid inappropriate drug use, to discontinue drugs that do not show benefits, and to avoid undertreatment with drugs that have shown benefits.	Education of staff
García-Talavera et al., 2012	Before-and-after	Patients with diabetes	A mixed intervention, with educational, self-evaluation and feedback components. Specific hospital admission critera due to acute diabetes decompensation were established. On the other hand, information brochures teaching the patient how to handle at home mild hypoglycemia and hyperglycemia were supplied as tools for the diabetologic education of the patient.	Educaion of patients

Garner et al., 2022	Before-and-after	Care home residents	HealthCall. Upskilling care home staff to use app-based technology whereby residents with new clinical presentations' observations are recorded electronically using a structured SBAR approach. Information is fed to a Single Point of Access where clinical staff triage the referrals.	Screening tool
Gellis et al., 2014	RCT	Medically frail older homebound individuals, aged 65 and older who were above-average users (≥10 days in the hospital in the past 12 months, seen in the emergency department (ED) in the last 2 months, or required ≥3 home care visits per week) and had a primary diagnosis of heart failure or COPD. Individuals who screened positive for depression as indicated by a Patient Health Questionnaire-2 (PHQ)16 score of 3 or greater were included in the study.	The I-TEAM (Integrated Telehealth Education and Activation of Mood) intervention consisted of integrated telehealth chronic illness and depression care, with a telehealth nurse conducting daily telemonitoring of symptoms, body weight, and medication use; providing eight weekly sessions of problem-solving treatment for depression; and providing for communication with participants' primary care physicians, who also prescribed antidepressants.	Telemonitoring
Ghimire et al., 2021	Before-and-after	Patients 18 years and older with frequent exacerbations of COPD and asthma	A single home visit. The home visit team comprised of the chronic lung program team that included nurse practitioner, respiratory therapist, medical social worker and outreach social worker. The team assessed medical history and medication use. Interventions included disease specific education, trigger assessments, and onsite spirometry. Social needs such as transportation barriers were assessed by the medical social worker.	Home visit
Giazzoni et al., 2023	Before-and-after	ED patients with a chief complaint of substance use and/or drug overdose	A REDO Case Manager (CM). If the CM was unable to reach the member within 24 hours, the member was referred to Community Paramedics (CP) to conduct a face-to-face home visit. CP utilized an intensive care management model providing support, care, and education that included naloxone distribution to decrease hospital readmissions and maintain optimal patient health.	Home visit and care coordination
Giles et al., 2023	Retrospective cohort	Patients with cirrhosis and an index hospital admission with decompensation (ascites, HE, jaundice, spontaneous bacterial peritonitis and/or variceal haemorrhage)	A nurse-led early postdischarge (EPD) clinic delivering goal-directed care for cirrhosis complications. Patients in the nurse- led clinic were seen face- to- face at each visit by one of two senior hepatology nurse specialists (NHS Agenda for Change Band 7). The consultations took place in a dedicated clinical area with access to a procedural suite for paracentesis if required. The nurses' input included nutritional advice and prescription of supplements and a specialist dietician could also be called if required by the nurses.	Follow-up program
Gingold et al., 2022	Non-randomized controlled trial	patients with low acuity calls to 911	Minor Definitive Care Now (MDCN). The MDCN field team consists of a community paramedic as well as an emergency physician or nurse practitioner, and uses a sports utility vehicle equipped for treatment in place. Monitoring 911 dispatch calls in real time, the MDCN team responds to low-acuity calls in parallel to standard emergency response. Faced with multiple eligible calls, the team may select calls that are amenable to avoiding ED transport based on caller location, complaint, age, or other dispatch system information. The MDCN team screens the patient and, if clinically appropriate and the patient consents, renders definitive on-scene care.	Prehospital triage and treatment
Gomis-Pastor et al., 2023	RCT	Heart transplant (HTx) recipients	mHeart strategy: multifaceted theory-based interventions were provided during the study period to optimize therapy management using the mHeart mobile application. At T0, T1 and T2 all patients received counseling by the pharmacist on how to improve medication self-management using behavioral theory-based treatments. These interventions were delivered using motivational interviewing.	Self-management

González-Ortega et al., 2016	RCT	Complex chronic patients (CCP) from three primary care teams	The intervention period lasted six months, and each patient in the intervention group received 11 phone calls (two weeks after the first face-to-face visit, they received twice monthly phone calls since complete the 6 months of follow-up). During each call, the intervening family physician asked patients about their current health status and symptoms, medication adherence, possible side effects of treatment, and the presence of social problems or any other contextual problem that might affect their chronic health conditions. If the patient had a caregiver, the physician talked also with them. The physician also reviewed the patient's electronic clinical record in order to ensure awareness of any clinical incidence regarding test results, visits to specialists, changes of treatment, hospital admissions or emergency visits.	Telephone follow-up with physician
Gould Rothberg et al., 2021	Before-and-after	Patients at the Oncology Extended Care Clinic (OECC) clinic	Opening of Oncology Extended Care Clinic (OECC)	Additional clinics
Grafstein et al., 2017	Before-and-after	"red" callers using a telephone triage service	The use of an emergency physician (EP) in a provincially nurse-staffed telephne service for urgent caller advice. The EPs spoke to "red" callers to provide further guidance.	Telephone triage (using an EP)
Grove et al., 2021	Retrospective cohort	Patients 18 years of age or older and received a diagnosis of schizophrenia or a schizoaffective disorder	Enhanced primary care that includes features tailored for individuals with serious mental illness (SMI): care coordination, peer support, and self-management programs and receives referrals of people with SMI who are receiving outpatient behavioral healthcare from community providers but are not in primary care.	Structural change in primary care
Guarnaccia et al., 2018	Before-and-after	All patients aged 6-17 who attended the Center	The diagnostic therapeutic educational pathway (DTEP) included 3 specialist's evaluations at 8- to 12-week intervals and two follow-up visits. Patients and their parents received an educational course concerning prevention measures, early recognition of symptoms, and appropriate use of drugs and devices.	Care program including follow-up and education of patient and parents
Haltia et al., 2021	Before-and-after	Patients aged 18 years or older who had incurable advanced cancer without further life-prolinging oncologic therapies	PC (palliative care) pathway. The patients had a palliative home care team responsible of the PC at home, if this was desired, and a planned ward in a community hospital for EOL care, where patients could be admitted from the ER or straight from their home according to their own wished at any time. A PC outpatient unit was established at the hospital. This included one part time physician with special competency in palliative medicine and a nurse. The PC unit coordinated the PC pathway and provided consultation serviced for the communities and hospital.	Care program
Harrap et al., 2022	Before-and-after	Patients referred to the LIMOS (Lewisham Integrated Medicines Optimisation Service) service. The LIMOS service can be accessed by any patient registered with a Lewisham GP where medicines support is required and one of the referral criteria are met.	Lewisham Integrated Medicines Optimisation Service (LIMOS). The service provides a formal pathway for the referral of patients with medicines-related problems identified in primary and secondary care for management by a specialist pharmacy team. The team, comprising of specialist pharmacists and pharmacy technicians, assesses, supports, and follows referrals with the aim of providing an integrated and deliverable pharmaceutical care plan. The aim is to enable patients, particularly those who are at high risk of admission, to get the most from their medicines whilst remaining independent and safe. The service is conducted in hospital, patients' homes and in care homes.	Pharmacist involvement
Hastings et al., 2019	RCT	Patients discharged from the Durham VAHCS (VA Health Care System) ED, received primary care at a Durham VAHCS-affiliated clinic, and were at high risk for repeat ED visits.	Telephone support which consisted of two core calls by a primary-care nurse in the week following an ED visit. The structured support focused on three key areas: (1) improving the transition from ED to primary care; (2) enhancing chronic disease management; and (3) educating Veterans and family members about VHA's primary medical home model and other VA and community services.	Telephone follow-up
Hawa et al., 2022	Prospective cohort	Children seen in the GI clinic for constipation at a tertiary care referral center	Constipation action plan (CAP): 3 intervention levels based on the patient's current symptoms. Each included instructions on behavioral and pharmaceutical treatment tailored to the patient's symptom severity as well as when to proceed to the next step for intervention.	Case management

Heath et al., 2015	Before-and-after	Patients discharged from a childrens hospital	Standardized follow-up phone calls by a physician after hospital discharge.	Telephone follow-up with physician
Hochman et al., 2013	Before-and-after	All patients visiting primary care internal medicine clinics	The patient-centered medical home PCMH: The PCMH model was desgined to (1) expanded access to care, (2) enhanced care coordination, and (3) team-based care. The intervention included the development of a call center, a process for renewing medication prescriptions by telephone, urgent care appointment availability, and enhanced case management.	Increased clinic access and care coordination
Holmes et al., 2020	Retrospective cohort	Homeless patients presenting to the study hospital clinics and ED during the study period	A program using a bricks and mortar dedicated homeless clinic, administrated by hospital clinical systems, staffed with family medicine physicians, located close to homeless shelters and camps at Fort Worth, TX, and functions as a medical clinic for this unique population.	Additional clinics
Howarth et al., 2016	Retrospective cohort	Patients discharged home from an ED following a CP presentation with a negative troponin measurement	Follow-up care cohorts: (1) Chest Pain Clinics (CPC; n=2 804), (2) specialist follow-up (SFU; n=4 046) with an Internist or Cardiologist without formalized coronary risk stratification testing	Follow-up program
Howell, 2016	Before-and-after	Medicaid and uninsured patients	Telephone triage 24/7. The initial staff consisted of a department director, 4 registered nurses (RNs) working 12-hour shifts and 2 on-call RNs as contingency staff. Marketing involved a refrigerator magnet, neighborhood events, radio ads, television interviews, and newspaper ads and by placing flyers in churches, as well as community-owned buildings. Further, they networked with local clinics and found some who were willing to provide standing appointments to be filled with telephone triage callers.	Telephone triage
Huang et al., 2020	Before-and-after	Patients with sickle cell disease > 18 years old	Individualized care plan. A care plan that tailors analgesics choices, dosage, fluid rate and non-opiate therapies for home, ED or inpatient ward settings. A copy of the care plan was given to the patient and scanned into Electronic Medical Records.	Case management
Hwang et al., 2018	Retrospective cohort	Uninsured patients ages 18 and older	Free clinics. They typically offer general medical care, immunizations, chronic disease management, pregnancy testing, gynecology/birth control, and links to medication assistance. Some clinics provide mental health counseling, sexually-transmitted disease testing and treatment, limited on-site dental care or referrals to regional dental clinics, social services, and health education.	Free primary care clinics
Haag et al., 2016	RCT	High-risk elderly (aged ≥60 years) who were enrolled in the local care transitions program (CTP).	A medication therapy management consultation with a pharmacist by telephone, preferably within 3 (and up to 7) business days after hospital discharge.	Telephone follow-up with pharmacist
Kalwani et al., 2015	Before-and-after	Frequent ED users	Community health workers (CHWs): these CHWs visit discharged patients and assist them in obtaining basic services, prescription refills and transportation to outpatient appointments.	Care coordination
Kapoor et al., 2020	RCT	Adults aged 18 and older with an episode of VTE diagnosed in the previous 2 weeks in hospital or ambulatory settings (office or ED) and prescribed warfarin, direct oral anticoagulant, or low molecular weight heparin.	A multicomponent intervention that included a home pharmacist visit in the week after randomization (typically occurring at time of discharge), illustrated medication instructions distributed during home visit, and a follow-up phone call with an anticoagulation expert scheduled for 8 to 30 days from time of randomization.	Home visit and follow-up
Kedan et al., 2016	Before-and-after	NYHA Class II-IV HF patients at high risk for hospitalization	The HF program included cardiologists and a specialty trained clinical pharmacist, who performed education, medication reconciliation, safety monitoring and guideline directed medication optimization under cardiologist supervision. Visits occurred every 2-6 weeks. Serial BNP and POC ultrasound measurements were used to guide HF management, the need for in-office IV diuretics and outpatient thoracenteses.	Care program

Kilburn et al., 2017	RCT	Families referred to the FBP (first-born program)	Intensive home visiting program delivered in homes of primary caregivers and their first-born children. The FBP home visitor team includes a registered nurse or other licensed health care professional, who provides a postpartum home visit, delivers the medical components of the curriculum, and continues to participate in the home visits when families encounter medical challenges. The second member of the home visitor team is a parent educator who generally has greater than a high school education and some human services experience. The parent educator delivers the nonmedical components of the curriculum	Home visit
Kim et al., 2022	Before-and-after	Multi-visit patients also known as “super utilizers”	The Complex Care Coordination (CCC) clinic. Each enrolled patient receives a comprehensive assessment of their medical and psychiatric history as well as their social determinants of health. Interventions include healthcare maintenance, psychiatric care, medication reconciliation, subspecialty referrals, substance use navigation, linkage to community resources, and housing/transportation/food resources.	Care coordination
Kim et al., 2023	Retrospective cohort	Children aged 3 to 17 years who received their primary care services at federally qualified health centers (FQHCs)	The Transforming and Expanding Access to Mental Health Care in Urban Pediatrics (TEAM UP) model, which is a comprehensive integrated mental health (MH) care model that serves children from birth through young adulthood. TEAM UP focuses on promotion, prevention, and early identification of emerging MH issues, swift access to MH services, and psychiatric consultation for complex cases.	Care program
Lachaud et al., 2021	RCT	Homeless adults with mental illness	HF (Housing First) involved immediate access to housing and mental health support services and included a monthly rent supplement (C$ 600) - and assertive community treatment (ACT). ACT included an inter-professional team offering nursing and medical care, case management support, and peer support with 24/7 coverage.	Care coordination and housing
Lai et al., 2022	Retrospective cohort	Hospital-discharged older Chinese adults aged 65+ with chronic conditions who were identified as high-risk patients of hospital admission and referred by public healthcare providers.	Home visits on a monthly basis were scheduled in 4 to 6 consecutive months, with follow-up telephone calls in between. Volunteers provided services in pairs based on individual preference or practical consideration such as mutual availability. Each pair was randomly matched to two elderly patients. The main roles of volunteers were to conduct systematic health assessments, provide educational health advice and emotional support, as well as acting as a key link person to enable the older adults to seek community resources, community nurses and social services if needed. The health check was performed during the home visits including measurement of blood pressure, body weight, body temperature, and blood oxygen saturation level if applicable.	Home visit
Latysheva et al., 2022	Before-and-after	Pediatric patients with uncontrolled Type 1 diabetes mellitus (T1DM)	Care Coordinator/Liaison who was designated to be a "personalized diabetes buddy". The CC contacted families for appointment reminders and participated in appointments to stay updated with clinical care plans and become a trusted link between clinical team and families. During hospital admissions the CC rounded with the diabetes educators who provided bedside education. CC set goals with patients and families.	Care coordination
Lee et al., 2016	Before-and-after	Pediatric gastroenterology patients	Pediatric gastroenterologists began receiving reports with rates of ED utilization by their patients. The reports contained information on only those patients who were seen in the ED and discharged from the hospital, excluding those who required admission to the hospital or to observation.	ED reports to specialists

Lee et al., 2017	Time series	Pediatric patients visiting specialist-physicians	ED utilization reports: the pediatric specialists began receiving reports with rates of ED utilization by their patients. Two separate reports were distributed: (1) 1 individualized to the physician; and (2) 1 with all physician rates for the divisional leadership. The divisions received follow-up reports every 6 months. In terms of specific interventions, this was left up to the divisions. The majority of the divisions did not have a specific intervention: neurology, hematology and oncology, and infectious disease. On the basis of their data, the pediatric pulmonologists implemented a call-back program in which their nurse would call high-risk patients after discharge from the hospital.	ED reports to specialists
Lin et al., 2015	Prospective cohort	Home-dwelling older patients aged ≥60 years admitted to the general medical wards of QMH (Queen Mary Hospital)	Integrated Care and Discharge Support for elderly patients (ICDS). For the hospital component, risk stratification, comprehensive geriatric assessment, and discharge planning were performed. Link nurses (who serve as 'link' between in-patients and community services) work with geriatricians to perform multidimensional assessments. In the community component: ICM (Integrated Care Model) Case Management and HST (home support team) service. Two social workers, one physiotherapist (PT), one occupational therapist (OT), and half a nurse (advanced practice nurse) take turns to be a case manager. THE HST includes nurses, PT, OT, and other allied health members.	Care coordination
Litzelman et al., 2017	Before-and-after	Medicare beneficiaries aged 65 or older with an International Classification of Diseases, Ninth Revision code of depression or dementia, who had at least 1 visit within the past 2-years to a primary care practice	Care coordinatior assistants (CCA) conducted and documented ACP (advance care planning) conversations with patients during home health visits. They work in interdisciplinary teams and provide care to patients and caregivers at home as part of Aging Research's Aging Brain Care (ABC) Program.	Home visit
Lukin et al., 2016	Controlled before-and-after	Residential aged care facilities (RACF) patients	The Hospital in the Nursing Home (HiNH) program operated mainly by a team of ED-based nurses. Major components of the intervention included sending clinical staff from hospitals to RACFs to provide outreach services for RACF patients, and providing support and education for RACF staff and GPs to increase their knowledge and confidence in the acute care for their patients, such that an increased range of procedures (e.g., urinary catheter change, parenteral antibiotic administration, wound care, etc.) could be alternatively provided within patients' own facilities instead of at EDs	Education of staff and outreach hospital
Marton et al., 2022	Retrospective cohort	Pregnant women	10-session CenteringPregnancy curriculum. These sessions include information on when to seek emergency care versus calling the office for an appointment (Sessions 1, 4, 5, 7, 8, and 10), managing common discomforts of pregnancy (Session 2), recognizing preterm labor (Session 4), and preparing for labor and birth (Sessions 5, 6, 8, and 10).Sessions begin with an individual routine medical examination for all women during the first 30 min and then transition to a 60- to 90-min facilitated group discussion led by a health care provider.	Education of patients
McAlister et al., 2018	Prospective cohort	all Albertans aged 20 years or older who were seen by a primary care physician at least once in fiscal years 2008 or 2009	A primary care network. In Alberta, primary care networks were established in 2005 to help facilitate both access to primary care and adoption of the Patient Medical Home model of care, which focuses on chronic disease management, health maintenance and prevention. There is no one structure for primary care networks (varying from one co-located clinic to several offices in a geographic area).	Structural change in primary care
McCarthy et al., 2017	Before-and-after	High healthcare utilizers. Adult patients (18 years or older) who have been referred to the community paramedic program and received at least one CP (community paramedic) visit or phone call	Community Paramedics (CP) program. CPs conduct home/shelter visits, review medication compliance, and arrange social services among other activities.	Community paramedic program

McCormack et al., 2013	Controlled before-and-after	Frequent ED users: at least 5 ED visits annually for 2 consecutive years and 1 within 6 months. Inclusion criteria were also had alcohol dependence, and had been undomiciled without shelter use for 9 of 24 months	On each subsequent visit, the social worker and outreach team met with participants, guided by previously developed care plans to offer shelter on discharge. Assigned caseworkers relocated participants into increasingly supportive settings, coordinated multidisciplinary care, and updated plans during biweeklyinteragency meetings on the basis of participants' medical, psychosocial, and housing needs.	Case management
McGuire et al., 2009	Before-and-after	Homeless veterans	Mental Health Outpatient Treatment Center (MHOTC) co-located in a newly renovated building with the offices of both the homeless social services programs and mental health programs. Homeless veterans were evaluated in a screening clinic and quickly referred to all needed services within the MHOTC building. The goal of the MHOTC was to have the initial primary care appointment occur the same day that the homeless veteran came to the screening clinic, i.e., the first day of arrival at the screening clinic. Policies, standard operating procedures, case conferences, and weekly building operational meetings were used to facilitate inter-clinic coordination and communication.	Co-location of clinics
McWilliams et al., 2019	RCT	Patients, under the care of a hospitalist, and at high risk for readmissions	Transition Services (TS). For 30 days following discharge to home, these patients had access to TS, which in addition to a free-standing clinic included (a) access to transition-dedicated internal medicine, pharmacist, paramedicine, behavioral health, and social work providers with scheduling frequency and provider type matched to patient needs; (b) hospital follow-up evaluation with a medical provider, either virtually in the patient's home facilitated by a paramedicine provider or in the transition clinic; (c) comprehensive medication reconciliation by a pharmacist; (d) at least weekly contact with a team member; and (f) coordinated transition to the next appropriate care location after 30 days.	Follow-up program
Melchiorsen et al., 2024	Before-and-after	Patients attending the orthopaedic A&E department at the Odense University Hospital with an injury	Mandatory referral practice (MRP). After the MRP was implemented, patients needed a referral to gain access to the A&E Department. Referrals are made by GPs or via help line calls manned by doctors and nurses or calls to the Danish emergency number 1-1-2.	Mandatory referral
Mierdel et al., 2015	Before-and-after	Patients with Chronic Obstructive Pulmonary Disease (COPD) and/or Congestive Heart Failure (CHF)	Telehomecare initiative brings together specially trained clinicians and technology to help patients with chronic disease monitor vital signs and learn to manage their health at home through self-management coaching. Oxygen saturation, weight, blood pressure and heart rate are measured weekdays and transmitted by tablet to a designated clinician who can monitor patterns.	Telemonitoring and self-management
Misra-Hebert et al., 2022	Retrospective cohort	Adult patients with a high risk score (≥40) at dicharge	A post-discharge home visit program utilizing the skills of advances practice nurses (APRNs) who are independent practitioners, and paramedics who have expertise in responding to acute care.	Home visit and follow-up
Mooney et al., 2021	Prospective cohort	Patients 18 years or older with cancer	Huntsman at Home (HH), a hospital-at-home program. Patients were admitted who required continued acute-level medical care after hospitalization or who had continuing unstable symptoms related to treatment or disease progression that would otherwise require ED evaluation or further hospitalization.	Home visit and follow-up

Morales et al., 2018	Controlled before-and-after	Cirrhotic patients discharged after being initially admitted for one of the following cirrhosis-related complications: hepatic encephalopathy (HE), ascites with or without renal failure, upper gastrointestinal bleeding (UGIB) or spontaneous bacterial peritonitis (SBP).	HEPACONTROL group received a close follow-up examination by a hepatology specialist at the Hepatology Unit Day Hospital within seven days after their discharge. Clinical evaluation in the follow-up visit included recording of vital signs and body weight and a physical exam. Patients were also interviewed to determine precipitating and risk factors for possible cirrhosis-related complications and to confirm compliance with medication and diet recommendations given at discharge. Patients were asked about bowel movements at home and the color and appearance of stools, if there was a relapse or a new decompensation appeared. Additionally, we performed a laboratory test (hemogram, coagulation, serum bilirubin, albumin, sodium, potassium and serum creatinine) on all patients at seven days after discharge.	Follow-up by physician
Morphew and Altamirano, 2017	Before-and-after	Children, aged 2 to 18 years, with high-risk persistent asthma	Breathmobile (BM) program. The intervention included reminder letters for tests and recommended immunizations, quarterly screening and monitoring by a disease management coordinator, quarterly educational paper mailings reviewing self-management techniques, and a list of self-management classes.	Care program
Nejtek et al., 2017	Before-and-after	Frequent utilizers >18 years old (been transported to the ED ≥ 4 times within a 1-year)	Mobile integrated health (MIH) program. The program delivered twice weekly in-home visits, provided health education coaching, performed routine health screenings, vital signs, phlebotomy, electrocardiograms, injections, wellness check-ups, medication management, and assessed home safety issues.	Home visit
Nene et al., 2020	Prospective cohort	Adult patients with IBD (inflammatory bowel diseases).	Rapid access clinic service (RAC). RAC visits consisted of fast-track evaluation of disease activity using clinical assessment and laboratory markers.	Rapid access to clinical services
Newman et al., 2017	Time series	High utilizers in patient-centered medical home (PCMH)	Care manager in a patient-centered medical home (PCMH). An early focus for the care manager was to develop a list of VIP patients who were high utilizers of care, defined as either having two or more admissions per year or 10 ED visits per year, and then developing and implementing a comprehensive care plan for each VIP patient.	Case management
Nguyen et al., 2021	Controlled before-and-after	Cancer patients (excluded leukemias and nonmelanoma skin cancers)	Oncology urgent care clinics (UCC) to treat common acute care needs.	Additional clinics
O'Brien et al., 2019	Time series	Patients at primary care providers (PCPs) whose patients' had high rates of use of the local hospital ED	The Navigation Hub comprised of a hospital-based nurse and a care coordinator from a community service agency who fielded questions (by telephone and email) from participating PCPs and/or their clerical support staff from 9AM to 5PM on weekdays. It aimed to facilitate timely access to specialty medical care, diagnostic testing, and community and hospital services, including intensive case management for patients with complex care needs. When PCPs contacted the Navigation Hub, they were informed services they may not have been aware of which may be more appropriate for their patients, and might be advised of services or specialists with shorter wait times.	Care coordination
Ohuabunwa et al., 2021	Before-and-after	Patients either hospitalized or had visited the ER up to three times within a 90-day period during the study period	TCM (transitional care model) utilizing community health workers (CHWs). The intervention was focused on increasing patient access to primary care with linkage to a primary medical home, promotion of acquisition of self-management skills through education, and coordination of care through the support of a CHW who followed the participant for a period of 12 months post-discharge.	Care program
Ong et al., 2024	Before-and-after	At risk patients with complex co-morbidities and high social care needs	Hospital-to-home (H2H) programs. The enrolment consisted of two home visits to the patient which could be conducted virtually if deemed suitable. During the home visits, other than following up on the medical issues, we would perform medical reconciliation, conduct Advance Care Planning conversations, and attend to social/financial needs by referring them to social workers or community partners where necessary.	Home visit

Patel et al., 2022	RCT	Participants with newly diagnosed advanced stages or recurrent solid and hematologic cancers	Community health worker (CHW)-led advance care planning and symptom screening. Within 1 week of study enrollment, intervention participants were assigned to 1 of the CHWs who delivered a structured program, as described in our prior studies, twice monthly over 6 months. The program comprised 3 telephone segments that included (1) education about goals of care and advance directives; (2) tailored guidance on how to engage in advance care planning (ACP) conversations with clinical teams and document advance directives; and (3) weekly proactive symptom screening using the ESAS.32 The CHWs also engaged participants 1:1 in open-ended discussions. The CHWs, as in our previous studies, reviewed symptom scores of 4 or greater or any scores that changed by 2 points from prior assessments with the supervising nurse, who conducted the appropriate clinical intervention.	Care program including education, screening, and advice
Patel et al., 2017	Before-and-after	Children with epilepsy	The 5 major interventions included the following: (1) beginning an established urgent epilepsy clinic (UEC) to improve access; (2) deploying a seizure action plan (SAP) to improve knowledge around home seizure management; (3) supporting proper dosing of abortive seizure medications via the electronic health record (EHR); (4) providing reminder magnets with instructions of use for abortive seizure medications; and (5) review of previous high utilizers of the ED for epilepsy care to address unique issues.	Care coordination
Podolsky et al., 2018	Before-and-after	Medically complex, homebound patients	Medical Care at Home piloted the use of community paramedics (CP) working directly with a telehealth physician for three types of visits: urgent, post-ED visit, and post-hospital discharge.	Community paramedic program
Post et al., 2021	RCT	Complex patients at high risk for ED use	Community-based case management. The intervention included two components: (1) identification of target patients with a predictive model; (2) triage to case management agencies and patient-centric case management.	Case management
Prater et al., 2024	RCT	Survivors of intentional and unintentional injuries aged ≥14 who were admitted to the University of Washington's Harborview Level I Trauma Center.	A mental health intervention: Patients received a patient-centered mental health screening, intervention, and referral procedure targeting the psychological sequelae of traumatic injury that occurred over the course of 6 months after the index injury admission. Patients randomized to the intervention condition were visited by a master in social work intervention team member at the bedside. The social worker asked about each patient's unique concerns and treatment preferences and scheduled ongoing times to meet/ call the patient during the initial days and weeks postinjury. The social worker developed a unique treatment plan specific to the preferences of each participant, and with patient consent incorporated family members/other supportive caregivers into postinjury treatment planning.	Follow-up with care coordination
Racine et al., 2009	RCT	all patients from 1 of 4 pediatric practices younger than 21 years presenting to a busy inner city PED	The intervention consisted of telephone contact with the patient, parent, or guardian originating from the primary care site within 72 hours of the PED visit to inquire about the follow-up status of the patient, schedule a primary care follow-up visit if necessary, offer brief counseling regarding the availability of after-hours telephone contact at the primary care site in the event of episodic illness, and provide specific advice regarding the appropriate use of the PED.	Telephone follow-up
Raffetto et al., 2014	Time series	Patients who were enrolled in one of the medical home pilots	Medical homes. The Los Angeles County Department of Health Services (LADHs) expand its primary care services through the creation of patient-centered medical homes - a team-based delivery model that provides comprehensive and coordinated medical care.	Structural change in primary care
Riar et al., 2016	Before-and-after	Patients with a diagnosis of either COPD or Asthma	During each visit patients were educated, with a focus on inhaler technique by a patient educator.	Education of patients

Rosenthal et al., 2013	Time series	Patients attending primary care practices	The Rhode Island Chronic Care Sustainability Initiative (CSI): Participating practices were required to obtain patient-centered medical home recognition from the National Committee for Quality Assurance (NCQA), track and share with each other and participating payers a set of standard clinical quality indicators, and have ongoing quality improvement activities. The NCQA recognition required that practices establish and document structural capabilities and protocols in 9 areas. Initially, the pilot practices focused their clinical quality measurement and improvement efforts on evidence-based screening and management of diabetes mellitus, coronary artery disease, and depression.	Structural change in primary care
Rousey et al., 2013	Before-and-after	Patients with advanced lung cancer	Home care visits , with an average of 12 visits per patient	Home visit
Rovner et al., 2023	RCT	Black individuals with diabetes after an ED visit	The intervention included race-concordant community health workers (CHWs), a clinical pharmacist, a diabetes nurse educator, and primary care physicians (PCPs). CHWs delivered six 90-minute in-home initial treatment sessions over 3 months followed by 3 booster sessions over the next 8 months (16.2% of all sessions were delivered remotely due to COVID). The CHWs delivered diabetes self-care education and behavioral activation (eg, goal-setting, actionplans) to increase participant adherence to recommended diets, physical activity, and medications to improve glycemic control.	Home visit with education
Ruchlewska et al., 2014	RCT	Adult outpatients diagnosed with psychotic or bipolar disorder who had experienced at least one psychiatric crisis in the previous two years.	Crisis plan. Two types of advance statement were used: (1) a crisis plan formulated by the patient with the help of a patient advocate (Patient Advocate Crisis Plan: PACP); and (2) a crisis plan developed together with the clinician (Clinician-facilitated Crisis Plan: CCP).	Self-management
Sanchez et al., 2015	Retrospective cohort	Patients discharged home from hospital (the family medicine service at the Boston Medical Center)	Pharmacist follow-up telephone call. Pharmacists conducting patient telephone call interventions utilized a standardized data collection form that included a review of current clinical status, a medication reconciliation process that included an evaluation of discharge and home medications, a reminder of follow-up appointments, and a discussion of what to do if a problem occurred (contact acute care office vs report to ED). Specifically, the medication reconciliation process involved asking patients to bring their medications to the phone to obtain an accurate list of the medications they were taking.	Telephone follow-up with pharmacist
Sathyanarayanan et al., 2021	Before-and-after	Frequent users (3 visits in 12 months prior to enrollment)	The case managers created care plans for each patient through weekly care team meetings. They arranged transportation for appointments, scheduled physician appointments, aided in refilling patients' medications, communicated continuousy with physicians, and arranged support services such as community, legal,substance abusem homeless shelter, and other health services.	Case management
Schamess et al., 2017	Before-and-after	Chronically ill patients with one or more disabling conditions	OSU Healthy at Home (OSUHH). The first visit consisted of a full history and physical, along with a psychosocial needs assessment that covered physical limitations; transportation barriers; patterns of emergency room and hospital use; family and community support; home health; and evaluation of physical safety, food and housing security. The interval and content of subsequent visits was determined by the HBPC provider, with the aim of addressing identified health and social issues. Visits were scheduled every 3 months at a minimum; but many patients received more frequent visits.	Home visit
Schickedanz et al., 2019	Non-randomized controlled trial	High utilizers	A telephonic social needs screening and navigation program. The 14-question social needs screener typically took 5–7 min to complete. If a patient screened positive for one or more unmet social need and was interested in help, the program associate performed a full intake assessment (10–15 min) for enrollment in social needs navigation.	Screening and social program

Schmidt et al., 2015	Before-and-after	Patients 18 years or older with a diagnosis of type 2 diabetes and uninsured or underinsured	A community health worker-led diabetes education program (DEP). The DEP program consisted of 2 initial 60-minute educational sessions and quarterly clinical assessments scheduled for 30 to 60 minutes for a maximum of 6 patient-contact hours over 12 consecutive months. During the 2 educational sessions, the CHWs educated DEP participants about diabetes and the importance of blood glucose control, medication adherence, diet, and exercise. In addition to the educational sessions, CHWs performed quarterly clinical assessments of HbAlc, blood pressure,weight, and foot condition (visual and monofilament as¬sessment). They also assessed self-management behaviorsand facilitated goal setting at each visit.	Education of patients
Schmidt-Mende et al., 2017	Cluster RCT	Patients aged older than or equal to 65 years	Educational outreach visits to primary care practices with feedback on prescribing and the development of a working procedure on MRs (medication reviews).	Education of staff
Schumacher et al., 2021	RCT	Older adults with chronic health conditions	Coleman's evidence-based Care Transitions Intervention®. A 30-day program utilizing coaches to conduct a hospital visit prior to discharge, a home visit, and three follow-up phone calls after hospital discharge. The coach helps patients develop disease self-management capabilities and communicate with providers. The program works with patients of all literacy levels through one-on-one review of each pillar during multiple sessions and assessment of patients' understanding.	Follow-up with self-management and care coordination
Seaberg et al., 2017	RCT	Patients frequently visiting the ED	Patient navigator (PN) program: a PN who worked with ED patients to review diagnoses and prescriptions, helped arrange follow-up appointments and transportation, and identified community resources that could help the patient. The PN performed these duties at the time of initial visit, at any subsequent ED visits, and at the telephone follow-up calls within 2 weeks and 12 months of the initial visit.	Care coordination
Serdarevic et al., 2023	Controlled before-and-after	Adults 18 years and over, with a documented opioid use disorder, and administered buprenorphine/naloxone or intramuscular dose of naltrexone (medications for opioid use disorder), while in the ED	Bridge clinics as transitional care programs. Referral to bridge clinic, a low-barrier transitional care clinic located within a walk-in urgent care clinic at John Peter Smith Hospital that provides wraparound services to patients with opioid use disorder.	Additional clinics
Shah et al., 2015	Prospective cohort	Patients of a geriatric practice that lives in senior living community	Health e-Access could be used when they had acute illness symptoms. It provides patient-to-provider, real-time, or store-and-forward telemedicine service. The extensive services available included video and audio communication, images, video clips, coun and 12 lead electrocardiograms. A telemedicine assistant facilitated the visit with the older adult patient.	Telemonitoring
Shyian et al., 2023	Before-and-after	Patients aged 18 years or older with sickle cell disease followed by the Edmonton Hemoglobinopathy Clinic and who presented to Medical Outpatient Unit	Patients experiencing early VOC (vaso-occlusive crises) symptoms were able to present to the Medical Outpatient Unit (MOU), an infusion clinic shared with other benign and malignant hematology conditions, to be assessed and receive treatment. Patients were able to schedule their own appointments and were encouraged to present early before they developed severe symptoms. They would be assessed by a nurse, who could then release orders from their personalized pain plan, which is a standing set of orders from an SCD clinic physician. Examples of orders include intravenous fluids and pain medications. Based on the severity of symptoms, patients could also be referred to the ED or for assessment by a physician or nurse practitioner.	Additional services

Siddique et al., 2012	RCT	Low-risk COPD (chronic obstructive pulmonary disease) patients	Education. A locally-developed educational brochure was mailed to patients in the education group. The content of the brochure included topics which were included in the face-to-face education we provided to high-risk COPD patients in our previous study. The brochure we mailed to low-risk patients included recommendations for smoking cessation, influenza and pneumococcal vaccinations, regular exercise, information about medications for COPD, and information about recognizing and treating COPD exacerbations (see the supplementary materials).	Education of patients
Simpson et al., 2023	Controlled before-and-after	Older adults aged 65 and older who were admitted to one midwestern ED or inpatient unit	A community paramedic referral program for patients 65 and older who were discharged home from the ED or hospital. CP provided home visits within 24-48 hours of receiving a referral. CP home visit components included: home safety assessment, physical assessment, cognitive screening, diet and medication compliance, non-acute community resource referrals.	Home visit
Skertich et al., 2022	Retrospective cohort	Patients less than 18 years of afe who underwent initial gastrostomy tube (GT) placement	Standardized Discharge Instructions Intervention. The SDIs consisted of an in-person educational session with a trained pediatric surgery nurse practitioner which lasted approximately 30 minutes. The session entailed teaching the patient (if appropriate) and care provider what a GT is, how to use it for feeding and medication administration, how to flush it, how to care for the GT site, indications for, when and how to vent it and what to do and how to replace the tube if the GT is dislodged. It also provided education on common problems which included leakage, redness around the site, and granulation tissue and what to do if they occur. A modified doll with GT was used during this session and caregivers had the opportunity to practice various elements of care using the doll. Finally, expected follow up was discussed and a written handout summarizing the teaching instructions was provided which also included the size of the GT or button, balloon fill volume (if button was placed), surgeon, date of surgery, and follow-up appointment date with office contact information.	Education of patients and parents
Spector et al., 2019	Before-and-after	Pediatric patients	The multidisciplinary, pediatric pain clinic focused on in this study is located at a single site on the health care system's main campus. The clinic provides specialized, pediatric pain management services including both traditional management options like medication, physical and occupational therapy, and mental health counseling, as well as holistic treatments ranging from relaxation techniques to acupuncture.	Additional clinics
Stergiopoulos et al., 2017	RCT	Frequent ED users: Adults (≥18 years of age) who had five or more ED visits in the past 12-months, with at least one visit for mental health or addictions	Brief case management: The Coordinated Access to Care from Hospital EDs (CATCH-ED) team. CATCH-ED case managers worked with participants over 4–6 months to first foster engagement and identify needs and goals, secondly to connect participants to needed community-based services, and finally to transition and transfer participant care to longer-term community services. As part of the program, case managers offered outreach and home visits, crisis intervention, supportive therapy, practical needs assistance and care coordination, aiming to integrate hospital, community and social care and improve continuity of care.	Case management
Street et al., 2018	Before-and-after	Patients who are vulnerable in the community, the majority of whom are elderly	The Acute Care Navigation service. It is a non-clinical service comprising of three care navigators who liaise and share information between acute and community-based health services, social and third sector groups.	Community program
Swankoski et al., 2023	RCT (post hoc analysis)	Primary care patients (veterans) at high risk for hospitalization	Primary care intensive management program in a patient-centered medical home: utilizes interdisciplinary care teams to comprehensively meet the complex care needs of patients at high risk for hospitalization.	Care coordination

Tadros et al., 2012	Before-and-after	Adult frequent users of health services (with ≥10 EMS transports within 12 months and others reported by prehospital personnel with significant recent increases in transports)	Resource Access Program (RAP). RAP employs EMS (emergency medical services) system surveillance, case management, and referral to identify and modify medical and psychosocial factors fueling repeated calls to 9–1-1. The RAP Coordinator contacts clients by phone or in person. She investigates factors underlying the excessive use of acute care resources for primary care conditions, including lack of transportation, social support, and health literacy. The RAP Coordinator interfaces with primary care physicians, homeless services agencies, street outreach teams, hospital social workers, case managers, and adult protective services personnel. On occasion, the RAP Coordinator conducts house calls accompanied by the San Diego Homeless Outreach Team. RAP clients receive education regarding appropriate use of EMS and are connected with resources including equipment, transportation, housing, social services, mental health services, and primary care. Follow-up care consists of telephone calls and in-person reminders for repeat access of EMS for nonemergent needs.	EMS care coordination
Takahashi et al., 2012	RCT	Older adults (greater than 60 years) with a high (>15) score on the Elder Risk Assessment Index (ERA) with high-risk for rehospitalization	Home telemonitoring. We utilized the Intel® Health Guide, which is a Food and Drug Administration-approved device, in the patient's home. The device had real time videoconference capability and peripheral devices (scales, blood pressure cuff, glucometer, pulse oximeter, and peak flow). Patients performed daily 5–10 minute monitoring sessions for symptoms and biometric information.	Telemonitoring
Taliaferro et al., 2023	Before-and-after	Patients with COPD	A service, referred to as the 340B PAP. The 340B PAP within the health system allowed patients to access medications at significantly reduced prices at the hospital-owned or contract pharmacy.	Medication service
Thompson et al., 2014	Before-and-after	Patients at high risk for hospitalizations	Initial work with a pharmacist- driven discharge medication reconciliation model for patients transferred to skilled nursing facilities (SNF) was followed up with a total care model in a controlled patient population with capitated payments much like an accountable care organization (ACO) model. Pharmacy worked with the hospital and clinics to further enhance the medical home model to focus on patient health. Pharmacists provide discharge medication reconciliation and patient follow-up within 3 to 5 days post-discharge with medication therapy management (MTM) visits.	Follow-up with pharmacist
Thomson et al., 2020	Prospective cohort	Patients with ascites and/or hepatic encephalopathy (HE) admitted to a hospital for any reason	Patients were given educational materials on ascites and/or HE. Patients were called after discharge by a coordinator to reinforce the educational materials, remind them of important follow up visits and/or labs, and assess acute needs.	Education of patients
Tinland et al., 2020	RCT	Homeless adults with severe mental illness (SMI)	Housing First (HF) program. Participants were assigned immediate access to independent housing and support from the Assertive Community Treatment team (social worker, nurse, doctor, psychiatrist and peer worker) (HF group).	Care coordination and housing
Tsai et al., 2018	Before-and-after	ED high-users and occasional users	Primary care intervention. The intervention consisted of 2 components, an adult primary care clinic on-campus, free of cost for uninsured patients under 200% of poverty. Another component of the intervention was to actively urge insured ED patients who were ED high-users or having a chronic disease/primary care–preventable condition, to either acquire a primary care physician (PCP) if they did not have one, or regularly visit their existing PCP.	Free primary care clinics
Tudorache et al., 2013	Before-and-after	COPD patients	A comprehensive medical education programme (1h/day/5days)	Education of patients
Vernon et al., 2019	Non-randomized controlled trial	Patients aged over 65 and registered at a GP that was a member of the Solihull Clinical Commissioning Group (CCG)	Telephone contact with patients -post-discharge. Two band 6 nurses attempted to contact the patients by telephone within 48 hours of discharge. When contact was made, patients were given the opportunity to discuss issues arising after discharge and were offered a home visit by one of the two nurses. The visits were an opportunity to explore issues identified in the telephone call and to further assess patient needs.	Telephone follow-up

Vohra et al., 2020	Retrospective cohort	Patients with acute decompensated heart failure	Within 3 days of discharge, Community health workers (CHW)s conducted an initial home visit with a standard assessment including vital signs and questions regarding well-being, weight management, symptoms, and medication adherence.	Home visit
Volner et al., 2022	Before-and-after	Aerodigestive patients under the age of 18	Aerodigestive clinic (ADC). The ADC at this institution consists of a bimonthly full day clinic regularly attended by pediatric otolaryngology, pediatric pulmonology, pediatric gastroenterology, pediatric speech language pathology and pediatric anesthesiology.	Additional clinics
Wartelle et al., 2022	Before-and-after	Patients receiving care in the ED	Opening of two new UCS (unscheduled Care Service) services. Facilities that can treat patients with low acuity conditions as an alternative to ED. These services are performed, for the most part, by general practitioners and nurses.	Additional clinics
Watts et al., 2016	Before-and-after	The veteran population with a Care Assessment Needs (CAN) score in the top 10 % of overall 3-month risk for morbidity or mortality and at least one acute care visit in the last 6 months.	Patient Intensive Management (PIM). The intervention, PIM care, is designed to support and extend the VHA primary care structure, which relies on a patient-centered medical home model. Our local PIM program is a team-based, shared-practice model that focuses on care coordination, interdisciplinary treatment planning, home visits, and health coaching.	Care coordination
Westberg et al., 2014	Non-randomized controlled trial	Patients aged >65 years with selected diagnoses identified as high risk for readmission	Comprehensive medication management that was provided face-to-face in the patient's primary care clinic within 2 weeks of discharge.	Medication management
Westgard et al., 2017	RCT	Patients with non-traumatic dental conditions (NTDC) who are without dental insurance or covered by Medicaid	Distributing a voucher for free, prompt dental care	Patient financial incentives
Whittaker et al., 2016	Retrospective cohort	Patient-initiated visits to emergency departments for “minor” problems.	Extended access in primary care: included evening and weekend opening and served both urgent and routine appointments.	Increased primary care hours
Williams et al., 2020	Before-and-after	Patients with systemic lupus erythematosus (SLE) - the top two percent of medically and psychosocially complex patients	An integrated care management program (iCMP). Nurses lead care coordination efforts for patients considered high risk for frequent emergency department (ED) visits and hospitalizations.	Care coordination
Woods et al., 2013	Before-and-after	Patients with a primary, secondary, or tertiary diagnosis of a circulatory or respiratory condition/diagnosis	Telemonitoring. Each patient in the program was scheduled to test one or more of their vital signs (e.g., blood pressure, heart rate, and oxygen saturation). Voice and text prompts guided the patient through the testing process. The test results were transmitted from the patient's landline or via wireless communication. A Web-based software system displayed the results for the agency nurse to review. A nurse contacted the patient to obtain more information when one or more of the patient's vital signs were outside the established parameters or when the patient did not transmit data at the predetermined schedule.	Telemonitoring
Worster et al., 2020	Comparative study (not clear)	Cancer patients	Multi-disciplinary supportive medicine program: The program systematically screened for biopsychosocial distress utilizing the National Comprehensive Cancer Center Distress Thermometer (DT) and the Problem Checklist (PC) to identify practical, emotional, spiritual and physical issues. Here, we document the impact of the supportive medicine program on outcomes of emergency department (ED) visits, hospital readmission, and non-billable touchpoints associated with patient navigation and resource referrals.	Care coordination
Yang et al., 2012	Before-and-after	Parents or caregivers to hosptialized pediatric patients	Post-discharge telephone calls. The content of the call followed a script to ensure consistency. During the call, the patients’ caregivers were asked how the children had been feeling since returning home, whether they had experienced any new or worsening symptoms, whether the caregivers understood how to provide their medications, whether the caregivers had any questions regarding follow-up appointments, the care the children had received during their recent inpatient stay or whether the caregivers had any other questions or concerns.	Telephone follow-up

Yoffe et al., 2011	Time series	Primary care pediatric patients	A 20- page booklet was given to the parents of all children age 10 and under with the instruction that the information it contained could help parents (1) provide non-emergency care for their children at home and (2) make better decisions about when emergency services may be needed.	Education of parents
Zwietering et al., 2023	Before-and-after	All new patients who visited the outpatient clinic geriatric medicine	The patients underwent a multifaceted medication review (i.e. evaluation by at least a geriatrician, and/or pharmacist and use of clinical decision support system)	Medication management
