# Supplementary material for: Non-emergency department (ED) interventions to reduce ED utilization: a scoping review
Source: BMC Emerg Med. 2024 Jul 12;24:117. doi: 10.1186/s12873-024-01028-4 (PMC11242019; doi:10.1186/s12873-024-01028-4)
Supplement: Supplementary file 3 — Additional file 3. Details of the included systematic reviews. [file 12873_2024_1028_MOESM3_ESM.pdf]

Additional file 3: Details of the included systematic reviews

Author, year	Population (as described by the SR authors)	Number and study design (as described by the SR authors) *	Interventions (as categorized and described by the SR authors)
Althaus et al., 2011	Frequent ED users greater than 16 years of age	11 studies (3 RCTs, 2 controlled trials and 6 noncontrolled before-and-after studies)	**Case-management**: 7 studies (1 RCT and 6 before-and-after studies. In these studies case management referred to coordination of health services on behalf of the patient by multidisciplinary teams composed of nurses, social workers, and physicians. Coordination tasks were allocated to a case manager, who guided the patient through the care process and provided social support. The intervention was not limited to the hospital, and often extended to the community)<br>**Case-management 'like'**: 1 study (1 RCT described an intervention that was not designated as case management; however, the study components were similar to those described in case management interventions, including individualized care plans made available to ED personnel, social worker or psychiatric evaluation, provision of an appointment with a primary care practitioner, and multidisciplinary case conferences)<br>**Intervention based on social worker evaluation and basic help**: 1 study (1 before-and-after study evaluated counseling on use of health care and social system by a social worker)<br>**Intervention based on the evaluation of coordination needs and care plan**: 1 study (1 before-and-after study described an intervention based on the evaluation of coordination needs and establishment of a care plan by the patient and their chosen coordination group)<br>**Print-out of case notes to emergency physician**: 1 study (1 RCT tested the provision of a printout of case notes from the patient's last 3 visits to the emergency physician)
Auger et al., 2013	Pediatric patients	14 studies (10 RCTs, 2 pre-post studies, 1 prospective matched case contro1, and 1 quasi-experimental)	**Discharge interventions**: 14 studies (1 case-control and 3 RCTs, assessed asthma education with follow-up, 1 RCT assessed a self-management asthma education program with a post discharge game, 1 RCT assessed enhanced asthma education, 1 pre-post study evaluated educational materials, adherence assistance, discussed "emotions of asthma", video, and tailored nursing interactions, 1 RCT assessed 6 monthly post discharge educational sessions on physiology, medication and where to seek advice, 1 quasi-experimental study in cancer patients conducted a frequent needs assessment, education, home visits, fever guidance, telephone consultation and manual for home care, 2 RCTs in the NICU assessed medical access strategies post-discharge, 1 pre-post study assessed practices including removal of discharge weight criteria, engagement of family and evaluation of home environment prior to discharge, and arrangement of home visits and follow-up, and 2 RCTs primarily evaluated early discharge with phone calls and home visits [and some other interventions])
Chartrand et al., 2023	Patients ≥18 years of age	50 RCTs (of these, 16 RCTs reported on ED visits)	**Transitional care programs:** 16 RCTs (interventions included a program to help with self-management of COPD; medical reconcilliation and counselling intervention; a patient navigator; weekly telephone support to help coordinate medical visits, using medications and self-manangement; COPD education program; individualized exercise program; education, nurse transfer letter, telephone advice for those admitted to hospital with heart failure; individualized care plans and information; self-management handbook and drug safety information for kidney transplant recipients; one-touch smartphone, BP monitor, medication dispenser, and a necklace emergency call button; comprehensive assessment, discharge letter and follow-up; written information; discharge planning and follow-up (2 RCTs); verbal information (2 RCTs))
Crawford et al., 2017	Adults	11 studies (6 before-and-after, 1 retrospective audit study, 2 quasi-experimental, 1 cohort, 1 comparative systems analyses)	**Walk-in centres**: 4 studies (3 before-and-after and 1 quasi-experimental - designated walk-in centres)<br>**GP cooperatives**: 7 studies (3 before-and-after, 1 retrospective audit study, 1 quasi-experimental, 1 cohort, 1 comparative systems analyses - the services were co-located or nearby to the ED and mainly operated out of hours to relieve the ED caseload)
Di Mauro et al., 2019	Adult frequent ED users	14 studies (7 RCTs, 4 prospective observational and 3 retrospective observational studies)	**Case-management**: 14 studies [of these 14 studies, case-management did not involve ED staff] (various components were included among the 14 studies: 8 created individualized care plans, 4 incorporated telephoning patients, 1 included motivational support, 4 studies facilitated contacts with healthcare providers and 2 studies organized group meetings with patient, 2 studies showed how patients enrolled in CM programs were immediately identified by the computerized system guaranteeing them appropriate treatments, 2 studies included home visits and ambulatory care, 2 studies guaranteed that homeless people receive an apartment through social services, and 1 study, patients were enrolled in treatment pathways defined by other departments)

Doshmangir et al., 2022	General adult population	64 studies (14 RCTs, 3 multicenter research trials, 2 quasi-experimental studies, 4 controlled before-and-after studies, 21 non-controlled beforeand-after studies (NCBA), 3 time-series studies, 3 case-control studies, 1 prospective cohort study, 1 longitudinal study, 6 retrospective cohort studies, 4 cross-sectional studies, and 2 studies with no study design description)	**Care plan:** 2 studies (2 before-and-after studies: one assessed an individualised care plan including health assessment, social support, problem-solving, empowerment, education, goal setting and mentoring, and the other assessed care plans that included social work assessment, directives to call pain team for the development of pain contract, radiologic studies, out-patient referral for speciality clinics, urinary toxicology studies, managed care referral, and psychiatric assessment)<br>**Case-management:** 31 studies (8 focused on case-management interventions outside of a hospital and 23 were hospital-based interventions. Of the 23, 12 focused on case management as an ED initiated or medical centre-based intervention for frequent hospital utilizers)<br>**Care coordination:** 2 studies (1 RCT assessed a hospital-based care coordination for high-risk older people - using an extra care bundle comprising of three interventions: medication counselling, enhanced discharge planning, and phone follow-up, and 1 cohort study examining a multidiscipline ED-care coordination program using a regional hospital information system capable of sharing patients' individualised care plans between ED providers)<br>**Utilization review:** 17 studies (this consists of several different review activities: pre-admission authorisation (prospective review), concurrent review (during the patient stay), retrospective review (relying on medical records), and prospective review).<br>**Clinical information system:** 4 studies (1 before-and after study reported on the use of health information technologies to identify the most frequently visiting patients and easy access to individualised care plans through the electronic medical record to all healthcare providers, 1 time series study evaluated a physician's order entry system, 1 RCT assessed a computer-generated informational message directed to physicians, and 1 RCT evaluated the impact of reminders following the introduction of a clinical information system that included a physician's order entry system)<br>**Physician profiling:** 1 study (a controlled before-and-after study evaluated physician profiling, descriphysbed in the study as a cost-containment strategy where patterns of health care provided by a practitioner or other provider (e.g., hospital) for the defined population are compared to other norms - profles of other physicians or practice guidelines - based on practice)<br>**Consultation:** 1 study (a RCT evaluated a mandatory radiology consultation whereby each radiology examination required prior approval)<br>**Discharge planning:** 5 studies (1 before-and-after study considered discharge planning using a risk screening tool, and 2 RCTs examined discharge planning based on individual patient needs, and a further 2 RCTs evaluated the effectiveness of case management provided after patients are discharged from the hospital)
Flores-Mateo et al., 2012	The general population (not specific conditions)	48 studies (10 RCTs, 18 quasi-experimental studies, 2 case-control studies, 4 times series, 6 cohort and 8 cross-sectional studies)	**Increasing the number of primary care centers or primary care physicians**: 10 studies (1 RCT, 4 quasi-experimental studies, 1 case-control study, and 4 cross-sectional studies)<br>**Increasing hours of access to medical services**: 9 studies (5 quasi-experimental studies, 2 time-series, 1 cohort, and 1 cross-sectional study - different models included GPs looking after their own patients after hours, commercial companies employing after-hours doctors, using non-hospital emergency department out-of-hours, and GPs from different practices forming a non-profit organization to provide care for their own patients out-of-hours)<br>**Telephone triage and advice services out-of-hours:** 6 studies (4 RCTs and 2 time series)<br>**Educational interventions without a non-educational component**: 6 studies (3 RCTs, 2 quasi-experimental studies and 1 case-control - interventions have not been described in detail by SR authors for all studies)<br>**Barrier interventions**: 17 studies (1 RCT and 7 quasi-experimental studies evaluated cost-sharing (i.e. out-of-pocket payments), 2 cohort studies and 2 cross-sectional studies examined copayments (i.e. flat fee), and 1 RCT, 3 cohort studies and 1 cross-sectional study evaluated gatekeeping (i.e. referral to access the ED)
Godard-Sebillote et al., 2019	Community-dwelling persons with dementia	17 studies (17 RCTs) (of which 7 reported data on mean number of ED visits)	Full details of interventions were not described. The 7 RCTs reporting on mean number of ED visits are described as follows:<br>**Self-management/case-management:** 1 RCT<br>**Comprehensive geriatric assessment:** 1 RCT<br>**Case management/self-management/use of information and communication technology/educational material and educational meetings (healthcare professionals' education)**: 1 RCT<br>**Self-management:** 2 RCTs<br>**Teams/case management/self-management/use of information and communication technology:** 1 RCT<br>**Case management/use of information and communication technology /teams:** 1 RCT
Hoot and Aronsky, 2008	Not defined	40 studies evaluated solutions to crowding (of these only 1 before-and-after intervention study is clearly presented and reports on ED visits as an outcome)	**Social interventions**: 1 study (1 before-and after study evaluated primary care referral, health education and counselling in frequent flyer patients)

Huntley et al., 2017	Older patients (greater than 65 years) with acute medical problems who were at risk of an unplanned admission	19 studies (10 RCTs and 9 nonRCTs)	**Paramedic practitioners/emergency care practitioners**: 3 studies (1 RCT, 1 controlled study and 1 case series with a historical control group - who received care in the community by these specialists)<br>**ED interventions**: 3 studies (1 RCT and 2 controlled studies)<br>**Community hospitals**: 2 studies (2 RCTs)<br>**Hospital-at-home services**: 11 studies (6 RCTs, 1 quasi-experimental study, 3 controlled studies, and 2 studies with a historial control) (but in many of these interventions, patients were admitted to the ED and outcomes tend to be hospital readmission - so not applicable to this SR)
Iovan et al., 2020	Adult ED super-utilizers in the US population	41 studies with 46 intervention sites (8 RCTs, 9 quasi-experimental studies, 24 pre-post studies representing 29 intervention sites) (the article states 43 studies, but this does not match references in a table)	**Case-management**: 13 studies with 18 intervention sites (3 RCTs, 4 quasi-experimental studies, 6 pre-post studies [with 11 sites] - evaluated 'traditional' case management)<br>**Case-management plus another intervention**: 3 studies (2 pre-post studies evaluated case-management plus medical care plans, and 1 pre-post study evaluated case-management plus care coordination)<br>**Medical care plan**: 7 studies (1 RCT and 6 pre-post studies<br>**Medical care plan plus another intervention**: 3 studies (1 pre-post study evaluated medical plan plus social support, 1 pre-post study evaluated disease management, and 1 RCT evaluated a community health worker in addtion to a medical care plan)<br>**Care coordination**: 2 studies (1 quasi-experimental and 1 pre-post study)<br>**Care coordination plus another intervention**: 4 studies (1 quasi-experimental study examined CC and case management, 1 RCT examined CC with case management and a medical care plan, 1 quasi-experimental study examined CC with community health work, and 1 pre-post study examined CC with telemedicine)<br>**Disease management**: 3 studies (1 RCT and 2 pre-post studies)<br>**Pain management**: 2 studies (2 pre-post studies)<br>**Other/undefined interventions:** 4 studies (1 RCT, 2 quasi-experimental studies and 1 pre-post study - these various evaluated an ED-based patient navigator, a mobile integrated health care intervention on ED transports, a supportive housing intervention on EMS use, and an undefined social intervention that did not asses ED utilization)
Ismail et al., 2013	Patients with low-acuity presentations who could be directed to other, more appropriate, care services or self-care, rather than A&E	28 studies (13 before-and after or interrupted time-series studies, 7 cross-sectional studies, 6 non-comparative case studies, 1 cohort study, and 1 non-randomised controlled trial) plus 6 SRs	**Telephone triage**: 9 studies (4 before-and after or interrupted time-series studies, 1 cross-sectional study, 3 non-comparative case studies, and 1 cohort study) plus 2 SRs - interventions ranged from national telephone triage lines (for example, NHS Direct in the UK) to local advice lines and telephone services embedded within GP cooperatives<br>**Walk-in clinics or minor injuries units**: 2 studies (1 before-and after or interrupted time-series and 1 cross-sectional study)<br>**Community health centres**: 2 studies (1 cross-sectional study and 1 non-comparative case studies)<br>**GP out of hours/GP cooperatives**: 11 studies (6 before-and after or interrupted time-series studies, 4 cross-sectional studies and 1 non-randomized controlled trial)<br>**Emergency nurse practitioner**: 1 study (1 before-and-after study examined emergency nurse practitioners in residential-care facilities providing first-line medical care for residents)<br>**'Various'**: 3 studies (1 before-and after or interrupted time-series and 2 non-comparative case studies) plus 4 SRs (details not described)
Kirkland et al., 2019	Patients seeking ED care	15 studies (10 clinical trials and 5 cohort studies) (of which 11 evaluated pre-hospital diversion interventions)	**Pre-hospital diversion**: 11 studies (7 clinical trials and 4 cohort studies. In one clinical trial, all emergency calls for non-serious conditions were passed on to a nurse/paramedic following ambulance dispatch who, with the aid of computerised decision support, assessed, triaged and provided advice to the patients, including asking patients triaged and not requiring an ambulance whether they still preferred ambulance transport. In one cohort study, low-severity calls were diverted from emergency call centres to nurse call centres, in which nurses used evidence-based protocols to provide patients' instructions and referrals to primary care providers or urgent care.<br>Nine studies assessed the impact of an EMS-based strategy: five assessed a ‘treat and release’ strategy in which paramedics assessed and treated low-acuity patients at the scene while four studies assessed strategies in which ambulance crews either diverted low-acuity patients to a minor injury unit (MIU), a community-based falls service, or transported intoxicated patients to a detoxification centre.
Kumar and Klein, 2013	Adult frequent ED users	12 studies (2 RCTs and 10 pre-post studies)	**Case-management**: 12 studies (2 RCTs and 10 pre-post studies: 6 described a multidisciplinary CM team, 7 (5) of which incorporated physicians as part of the CM team, 2 used a single case manager, 9 studies reported using substance abuse counseling or referral services as part of their intervention, 7 studies reported assistance with financial entitlements, 7 studies reported using individualized care plans, and 3 studies reported using assertive and persistent outreach to assist patients in going to their appointments)

Leduc et al., 2020	Older adults in long-term care	22 studies (4 RCTs and 19 observational studies) of which 13 are reported to have presented data on ED visits, but results are only presented for 4 studies	**Use of advanced nurses or nurse practioners:** 1 study presented data on ED visits (study design not reported) (in this study, nurse practioners and physician assistants assessed patients through telemedicine to determine if they required a transfer to the ED or if they could be treated on-site)<br>**Asessment and treatment toolkit:** 1 study presented data on ED visits (study design not reported) (a program called Interventions to Reduce Acute Care Transfers (INTERACT) which consisted of a set of 7 tools aiming to prevent hospital admissions by identifying and treating changes in conditions early, managing conditions in the long-term centre when possible and improving advanced care planning. It also included an advanced practice nurse to provide direct care to patients)<br>**End-of-life care:** 1 study presented data on ED visits (study design not reported) (involved palliative care consults provided by nurse practitioners)<br>**Extended care paramedics:** 2 studies reporting on one program (study design not reported) (this program involved the use of extended care paramedics who responded to calls for acute issues in long-term-care centres)<br>In addition, this SR reported on acute treatment of specific conditions, but clear results for ED visits were not reported or were self-reported.
Moe et al., 2016	Adult frequent ED users	31 studies (6 RCTs and 26 before-and-after studies)	**Case-management**: 18 studies (case management involved multidisciplinary teams, including physicians, nurses, psychologists, social workers, and/or housing and community resource liaisons, who developed tailored care strategies for patients and linked them to necessary services)<br>**Care plans**: 8 studies (care plan interventions involved multifaceted patient health and social assessments to develop individualized plans to guide future care-givers)<br>**Diversion strategies**: 3 studies (diversion strategies sought to redirect appropriate patients to non-ED settings for care)<br>**Printout case notes**: 1 study (nvolved study staff printing out information from patients' previous three ED visits for the current physician providing care)<br>**Social work visits**: 1 study (social work home visits to identify patient needs)
Morgan et al., 2013	Not defined	39 studies (5 RCTs, 2 pre-post studies, 11 cohort studies, 16 before-and-after studies, 1 retrospective study, 2 time series, 1 quasi-experimental controlled trial and 1 cross-sectional study)	**Patient education**: 5 studies (2 RCTs, 2 pre-post studies and 1 retrospective cohort - involved booklets or in-person training sessions)<br>**Creation of additional capacity in non-ED settings**: 10 studies (2 before-and-after studies, 1 retrospective study and 1 cohort examined interventions that expanded capacity through new community clinics, and 6 before-and-after studies involved existing physician practices expanding appointments and/or hours of care)<br>**Managed care:** 12 studies (2 time series studies, 1 before-and-after study and 3 cohort studies had interventions with capitated payment of primary care physician, and 1 RCT, 2 before-and-after studies and 2 cohort studies had a requirement of primary care physician approval or gatekeeping, and 1 before-and-after study was a hybrid of these two)<br>**Prehospital diversion:** 2 studies (1 RCT involved transportation of such patients to clinic care without home care as an option and 1 cohort study involved EMS offering either home or clinic care to low-acuity patients)<br>**Patient financial incentives**: 10 studies (1 RCT, 1 quasi-experimental controlled trial, 3 before-and-after studies and 2 cohort studies, the intervention was the requirement for patient copayment or coinsurance, and in 1 cohort, 1 before-and-after study and 1 cross-sectional study - it was the implementation of a high deductible)
Morley et al., 2018	Adults or adults and children (but not studies of children)	102 studies (of these, 5 were intervention studies outside of the ED that evaluated ED attendance: 3 cohort studies, 1 time series and 1 case control)	**Social interventions**: 1 study (1 retrospective cohort study evaluated public education campaign on proper use of the ED, financial disincentives, redirection, and provision of alternative clinics)<br>**Walk-in centre**: 1 study (1 retrospective cohort examined a GP led walk-in clinic with opening hours from 8 to 9 pm, 7 days a week)<br>**After-hours GP**: 3 studies (1 restrospective time series evaluated a user-pays after hours GP clinic, 1 retrospective case control piloted a 7-day of GP practice, and 1 retrospective cohort also investigated extending GP opening-hours)
Morrison et al., 2013	Children aged 0 to 18 years and their parents	8 studies (3 RCTs and 6 nonRCTs [not defined])	**Parent education**: 8 studies (2 RCTs and 2 nonRCTs utilized asthma-specific educational interventions variously involving one or more components of home visits, education (courses), an environmental intervention [not defined], neigbourhood education or coordinated care, and 1 RCT and 3 nonRCTs utilized general pediatric health educational interventions involving health aid books)

Poku and Hemingway, 2019	Children aged 0 to 18 years presenting to an ED for non-urgent care	6 studies (4 RCTs and 2 quasi-experimental) (only two studies were not ED-based)	**Informational support**: 1 study (1 RCT was based in primary care and involved telephone contact with participants 72 hours after index non-urgent PED visit to inquire about follow-up and schedule a primary care follow-up if necessary, provide counselling on appropriate paediatric ED use and availability of after-hours services at primary care sites)<br>**Initial ED-based (discharge procedure) followed by a community follow-up**: 1 study (1 quasi-experimental study involving educating participants on the relevance of primary care and preventative care. Those without PCP were assisted in choosing and registering with one and provided with information on PCP office hours, the scope of practice, after-hour services and availability of public transportation and parking. They were assisted in scheduling a PCP follow-up appointment and followed up to determine compliance. Participants in the minimal intervention group were followed up by a clerical worker while those in the case management group were followed up by a paediatric nurse or a paediatric social worker for 3 months after index PED interaction and provided with in-depth information concerning potential barriers to primary care)<br>**ED-based interventions**: 4 studies (3 RCTs and 1 quasi-experimental studies)
Pulcini et al., 2021	Children with medical complexity	15 studies (2 RCTs, 13 retrospective cohort studies)	**Outpatient care**: 15 studies (2 RCTs and 13 retrospective cohort studies) involved care coordination as the central component of the intervention, nearly all studies used a multi-disciplinary approach, 8 studies also included individualized or emergency care plans, 7 studies also included access to known providers for 24 hours every day of the week by phone for families, and 4 studies also offered expedited or next day appoiontments)
Raven et al., 2016	Adults or adults and children (but not studies of children)	13 studies (5 RCTs, 1 quasi-experimental and 7 before-and-after studies)	**Case management**: 3 studies (1 RCT and 2 before-and-after studies targeted high-risk patients with frequent use of the ED and in all 3 interventions, case managers provided intensive direct services within the ED, hospital, and community by frequent, in-person contact with patients)<br>**Care coordination plus another intervention**: 3 studies (3 RCTs targeted low-acuity patients: one involved a telephone-based intervention for patients with annxiety disorder, in another, the intervention involved patient 65 years or older meeting with a geriatric nurse, who conducted a needs assessment during the ED visit, sent a summary to the patient’s primary care provider, and conducted telephone follow-up to encourage the primary care provider visit, while the last study focused on patients with no primary care and evaluated the effect of using ED-based health promotion “advocates” to help patients choose a primary care provider during the visit and then faxed the patients’ information to the chosen primary care provider. Advocates contacted patients after the visit in person or over the telephone to help schedule a primary care provider appointment and to connect patients to other community-based services)<br>**Asthma education program**: 1 study (1 RCT concerned adult and pediatric patients with asthma. The intervention consisted of a telephone call from an asthma nurse educator 3 to 5 days after the ED visit, who arranged and attended a primary care provider follow-up visit and created an asthma care plan for the patient. This nurse conducted a home visit 6 weeks later to evaluate environmental triggers and to review the care plan)<br>**ED diversion**: 1 study (1 quasi-experimental study invoved referring low-acutiy patients to an onsite primary care clinic (intervention) or to an ED-based urgent care clinic (usual care)<br>**Finanical penalties**: 5 studies (5 before-and-after studies examined ED copayments at the visit)
Rennke et al., 2013	Adult general medical patients	47 studies (28 RCTs and 19 controlled clinical trials) (of these 26 reported on ED visits)	**Transitional care' strategies**: "The most commonly used interventions included patient engagement (n=37), ranging from general patient education to more specific instruction on symptom management and medication counseling. Twenty-eight studies included postdischarge outreach to patients by telephone (n=10), home visit (n=8), or both telephone contact and at least 1 home visit (n=10)."
Stall et al., 2014	Homebound community-dwelling older adults	9 studies (1 RCT and 8 observational studies) (of these 4 reported on ED visits)	**Home-based primary care**: 4 studies (4 before-and-after studies)
Tricco et al., 2014	Adult frequent users of health care services	36 studies (36 RCTs) - of these, 6 RCTs evaluated ED visits	**Care coordination**: 6 RCTs (that reported on ED visits) evaluated interventions involving one or more of the following: case management, team changes, self-management or clinical information systems. 3 of these RCTs also included patient education
Wasan et al., 2024	Adults (greater than 18 years of age) with multimorbidity, defined as having at least two co-existing chronic medical conditions	13 studies (4 RCTs, 7 prospective observational studies, 1 retrospective cohort study and 1 cost-utility analysis)	**Care plan:** 10 studies (4 RCTs, 5 prospective observational studies, 1 retrospective cohort study). 9 of the care plans involved integrated care comprising of one or more of self-management, telemonitoring, follow-up, education, coaching or consultation, goal-setting, or home visits.<br>**Care coordination:** 2 studies (1 prospective observational study involved improved coordination between health providers and home-based care for patient through baseline assessments, follow-ups and patient empowerment programand; and 1 cost-utility analysis based on a cluster RCT that evaluated improved communication between primary and secondary healthcare professionals with support of a reference internist and liaison nurse who also provided educational support)<br>**Palliative care:** 1 prospective observational study evaluated a home visit after hospital discharge, followed up with scripted phone calls at 4 and 8 weeks to address any updates in patient status).

Weeks et al., 2018	Community-dwelling adults age 60 and older with at least 1 medical diagnosis	23 studies (19 RCTs and 4 comparable cohort/case control studies) of which 13 reported on ED usage (14 reported in text, but results presented for 13)	**Transitional care' programs**: 13 RCTs which reported on ED visits ("Transitional care services generally began with a baseline assessment to identify participant needs, and care was largely tailored to meet individual needs. Common supports provided included: coordinating and facilitating care across settings and care providers; support with accessing health and community services; providing information and education; health monitoring; health management and intervention; physical and environmental assessments; medication support; help with navigating the health system; supporting empowerment, autonomy, and self-management to help participants better manage their own health; encouraging the patient and caregiver to assert a more active role during care transitions; and support for unpaid caregivers." Nurses were most commonly identified as the providers)

* Controlled before-and-after and non-controlled before-and-after have been grouped
